# Physical and Psychological Factors Associated With Walking Capacity in Patients With Lumbar Spinal Stenosis With Neurogenic Claudication: A Systematic Scoping Review

**DOI:** 10.3389/fneur.2021.720662

**Published:** 2021-09-09

**Authors:** Mariève Houle, Jean-Daniel Bonneau, Andrée-Anne Marchand, Martin Descarreaux

**Affiliations:** ^1^Department of Anatomy, Université du Québec à Trois-Rivières, Trois-Rivières, QC, Canada; ^2^Department of Chiropractic, Université du Québec à Trois-Rivières, Trois-Rivières, QC, Canada; ^3^Department of Human Kinetics, Université du Québec à Trois-Rivières, Trois-Rivières, QC, Canada

**Keywords:** lumbar spinal stenosis, neurogenic claudication, walking capacity, gait pattern characteristics, functional task

## Abstract

**Objective:** The purpose of this study was to evaluate the current state of scientific knowledge regarding physical and psychological factors associated with walking capacity in patients with lumbar spinal stenosis (LSS) with neurogenic claudication.

**Design:** Systematic scoping review.

**Literature Search:** We searched CINAHL (Cumulative Index to Nursing and Allied Health Literature), MEDLINE, Cochrane, PsycINFO, and SPORTDiscus databases.

**Study Selection Criteria:** Cohorts and cross-sectional studies reporting on associations between physical or psychological factors and impaired walking capacity in patients with symptomatic LSS were included.

**Data Synthesis:** Data were synthetized to identify associations between physical or psychological factors and either walking capacity, gait pattern characteristics, or functional tasks.

**Results:** Twenty-four studies were included. Walking capacity was significantly correlated with several pain outcomes, disability, estimated walking distance, and cross-sectional area of the lumbar spine. Gait pattern characteristics such as speed and stride were strongly and positively correlated with disability outcomes. Functional tasks were significantly correlated with lower back and upper limb disability, lower limb endurance strength, ranges of motion, and speed. Associations with psychological factors were mostly conflicting except for the Rasch-based Depression Screener and the Pain Anxiety Symptom Scale (PASS-20) questionnaire that were associated with a decreased performance in functional tasks.

**Conclusion:** Physical and psychological factors that are associated with walking capacity in patients with symptomatic LSS were identified. However, many associations reported between physical or psychological factors and walking capacity were conflicting, even more so when correlated with walking capacity specifically.

## Introduction

Symptomatic lumbar spinal stenosis (LSS), characterized by a limited walking capacity due to leg pain, is a leading cause of disability in the elderly (1, 2). Narrowing of the lumbar spinal canal or lateral foramina, as well as compression or decreased blood flow to the nerve roots (3, 4) are considered the main causes of pain in this musculoskeletal condition. Lumbar spinal stenosis is affecting between 11 and 39% (5, 6) of the global population, especially people over the age of 65 (6, 7). The acquired form of LSS arises from degenerative changes, including disc degeneration (herniation or bulging), hypertrophy of the ligamentum flavum, and spondylolisthesis and/or facet osteoarthritis (3, 4, 8) and can involve the central canal, lateral recess, foramina, or any combination of these anatomical sites (8). Congenital LSS is due to abnormalities during development, leading up to smaller pedicles length, which directly affects the antero-posterior diameter of the spinal canal (9).

In patients with LSS, self-reported symptoms combined with a physical examination represent the main assessment components of the clinical portrait severity, considering that symptoms and associated disability do not always correlate with results from magnetic resonance imaging (MRI). Advanced imaging provides information on the presence and extent of lumbar spine degenerative changes and on the lumbar spinal canal size, but does not translate information on functional capacity (6). Indeed, a number of patients will show clear and severe signs of stenosis on imaging but will experience few or no symptoms. Commonly reported symptoms of LSS include leg pain, numbness, cramps, fatigue, and weakness (8, 10). These symptoms can be grouped under one appellation: neurogenic claudication (NC). The presence of NC is modulated by the patient's posture, as it is brought on by lumbar extension when standing or walking and relieved by lumbar flexion (i.e., sitting or bending forward) (8).

Neurogenic claudication is triggered by performing daily activities that require prolonged standing postures such as walking, and patients often face a decrease in quality of life (QoL) due to important walking and functional limitations. The assessment of walking limitations plays a central role in LSS management, both in the decision-making process regarding the diagnosis (11) and treatment options (12). Most recent studies evaluating treatment effects in patients with LSS have measured walking abilities using different assessment methods. Some of these studies focused on walking distance (13–15), while others focused on walking time (16, 17), or both (18, 19) to report on walking capacity. Indeed, severe symptoms combined with a decrease in walking capacity, and subsequently in QoL, prompt neurosurgeons to opt for surgery in patients with LSS (20, 21).

Walking impairment is a major issue in LSS, and many physical and psychological factors are related to this decrease in functional capacity. Several gait measures such as step length, cadence, step width, and gait cycle have been assessed in LSS patients, but it is not clear if and how they relate to the decline in walking capacity. It is also known that walking limitations can negatively influence or be influenced by psychological factors such as a perceived low QoL and self-efficacy, and increased anxiety and kinesiophobia (22, 23). Assessing physical and psychological factors may provide relevant information that would improve our understanding of how LSS affects daily activities.

The purpose of this study was to evaluate the current state of scientific knowledge regarding physical and psychological factors associated with walking capacity in patients with LSS and associated NC.

## Methods

As systematic scoping reviews are used to inform future research directions, this study design was deemed the most appropriate to capture information from heterogeneous studies, map existing literature and identify knowledge gaps (24). This scoping review was based on the framework of Levac et al. using a 5-step review method (25) and on the framework of Peters et al. for the systematic aspect of the study conduct (24). This study was registered on Open Science Framework (https://osf.io/6az7c/?view_only=15ad45d1f1f14517b3706b154af12bb6).

### Step 1: Identifying the Research Question

This scoping review was conducted to answer the following question: What are the physical and psychological factors associated with walking capacity in patients with LSS and associated NC?

The main focus of this systematic scoping review was walking capacity in patients with LSS and associated NC. Exploring associations between physical or psychological factors and walking capacity or performance in functional tasks should provide critical information regarding the impact of LSS on daily functioning. Interventions targeting physical and psychological factors associated with the decline in walking capacity could improve patients' walking capacity and QoL and support surgical decision-making.

### Step 2: Identifying Relevant Studies

The search strategy was elaborated in collaboration with a university librarian and was conducted using CINAHL (Cumulative Index to Nursing and Allied Health Literature), MEDLINE, Cochrane, PsycINFO, and SPORTDiscus databases from inception to October 4, 2019. Then, an update of the literature was completed on June 18, 2020. A combination of keywords and MeSH terms was used to identify relevant studies. The lead investigator (MH) conducted the literature search. The search strategy was first developed for MEDLINE and adapted to other databases when needed (see Supplement Material 1). Other sources such as Google Scholar and reference lists of relevant studies were hand-searched to ensure a comprehensive overview of the subject. An EndNote library (version X9, Clarivate Analytics, Boston, MA, USA) was created to import all citations from the search strategy. Then, all duplicates were identified and removed.

### Step 3: Study Selection

#### Definitions of Key Concepts

Symptomatic LSS was defined as back and/or leg pain causing NC in patients diagnosed with degenerative LSS. The targeted outcome was walking capacity which was considered from two different perspectives: walking capacity defined as a distance or time spent walking, and walking capacity defined as performance during a functional task that repetitively involve lower limbs and/or trunk movement (e.g., the Timed Up and Go test or stairs climbing). Associated factors of walking capacity were regarded as either physical or psychological. Physical factors were divided into patient-reported outcome measures (PROMs) [e.g., pain, disability and QoL] and objectives measures [e.g., gait pattern characteristics (e.g., speed, cadence, and step width), lower limb strength and range or motion (ROM)] while psychological factors of interest included anxiety, depression, kinesiophobia, frailty, and self-efficacy.

#### Inclusion and Exclusion Criteria

To be included in this scoping review, studies had to be limited to human participants and be published in peer-reviewed scientific journals in either French, English, or Spanish language. Study designs were limited to cohort, and cross-sectional studies. Randomized controlled trials were also considered provided that they present baseline data of participants with LSS and associated NC and reported on correlation or regression prior to the beginning of the intervention. In addition, assessments had to include at least one physical and/or psychological outcome measures. To be include, studies needed to fulfill inclusion criteria regarding the specific populations (P), intervention (I), and outcomes measures (O) that are presented in Table 1. In addition, studies that included participants with congenital LSS, scoliosis, or vascular claudication were excluded. The following types of publication were also excluded: validation study, case study, cases series, systematic review and meta-analysis, gray literature, and governmental documents.

**Table 1:** Inclusion criteria regarding population, intervention, and outcomes measures.

**Inclusion criteria**	**P**	**I**	**C**	**O**
	•Patients with LSS: Localization: - Central - Foraminal - Lateral •With or without spondylolisthesis •Coexisting LSS types (e.g., central + foraminal) •Presenting with NC •Having at least 18 years old	•Associations provided before any medical intervention is initiated •Data collected during one of these situations: - Walking - Climbing stairs - Running - Functional tasks	•Non-applicable	•Physical and psychological factors related to: - Walking capacity - Functional tasks - Gait pattern characteristics

*If there was a mixed sample (e.g., LSS and healthy participants), the study was kept only if data from patients with LSS could be extracted separately.*

*P, Population; I, Intervention; C, Comparison; O, Outcome measures; LSS, lumbar spinal stenosis; NC, neurogenic claudication*.

#### Screening

All potentially eligible articles were independently screened by a pair of reviewers (MH, MERP) in two phases, using a standardized Excel spreadsheet. In phase one screening, titles and abstracts were classified as relevant, possibly relevant, or irrelevant according to the eligibility criteria. Then during phase two screening, the full text of possibly relevant articles was reviewed by the same pair of reviewers for final determination of eligibility. Reviewers discussed disagreement to reach consensus for both phases of screening and a third independent reviewer (AAM) was consulted to achieve consensus if needed.

### Step 4: Charting the Data

The following descriptive variables were extracted from all relevant studies using a standardized extraction form: authors, year of publication, title, country, study design, sample size, definitions of both LSS and NC, description of the study population (number of participants with LSS, LSS type, age, and gender ratio), independent variables, dependent variables, and key findings from study results. A pair of researchers independently extracted data (MH, JDB) and if necessary, a third person (AAM) was involved to resolve disagreements.

### Step 5: Collating, Summarizing, and Reporting Results

#### Study Designs and Participants

Data regarding study designs, sample sizes, and patients' characteristics were summarized to provide an overall picture of the populations studied.

#### Quality Assessment

Quality assessment of all eligible studies was independently completed by two reviewers (MH, JDB) using the Appraisal tool for Cross-sectional Studies (AXIS) (26). When a disagreement between the two reviewers occurred, a third person (AAM) was involved to reach consensus. The AXIS tool contains 20 questions that address three different domains: study design (7 questions), study quality (7 questions) and risk of bias (6 questions). Each question is answered by either “yes” (1 point), “no” (0 point), or “do not know” (0 point). A sum of all “yeses” is calculated to provide an overall score with higher scores indicating higher quality. As none of the studies reported a description of the non-responders (question 7), question 14 (“If appropriate, was information about non-responders described?”) became non-applicable and therefore was removed from the quality assessment checklist, bringing the maximum possible score to 19. Furthermore, question 19 (“Were there any funding sources or conflicts of interest that may affect the authors' interpretation of the result?”) was given one point when it was specified that there were no conflicts of interest.

#### Results Organization

Results were first organized into two broad categories based on the nature of the reported associations. The categories were defined as follow: associations in relation to [1] walking capacity, and [2] functional tasks. Then, within each category, results were organized based on outcome measure types (i.e., PROMs, objective outcome measures, and any other relevant outcome whenever applicable).

## Results

**Figure 1:**
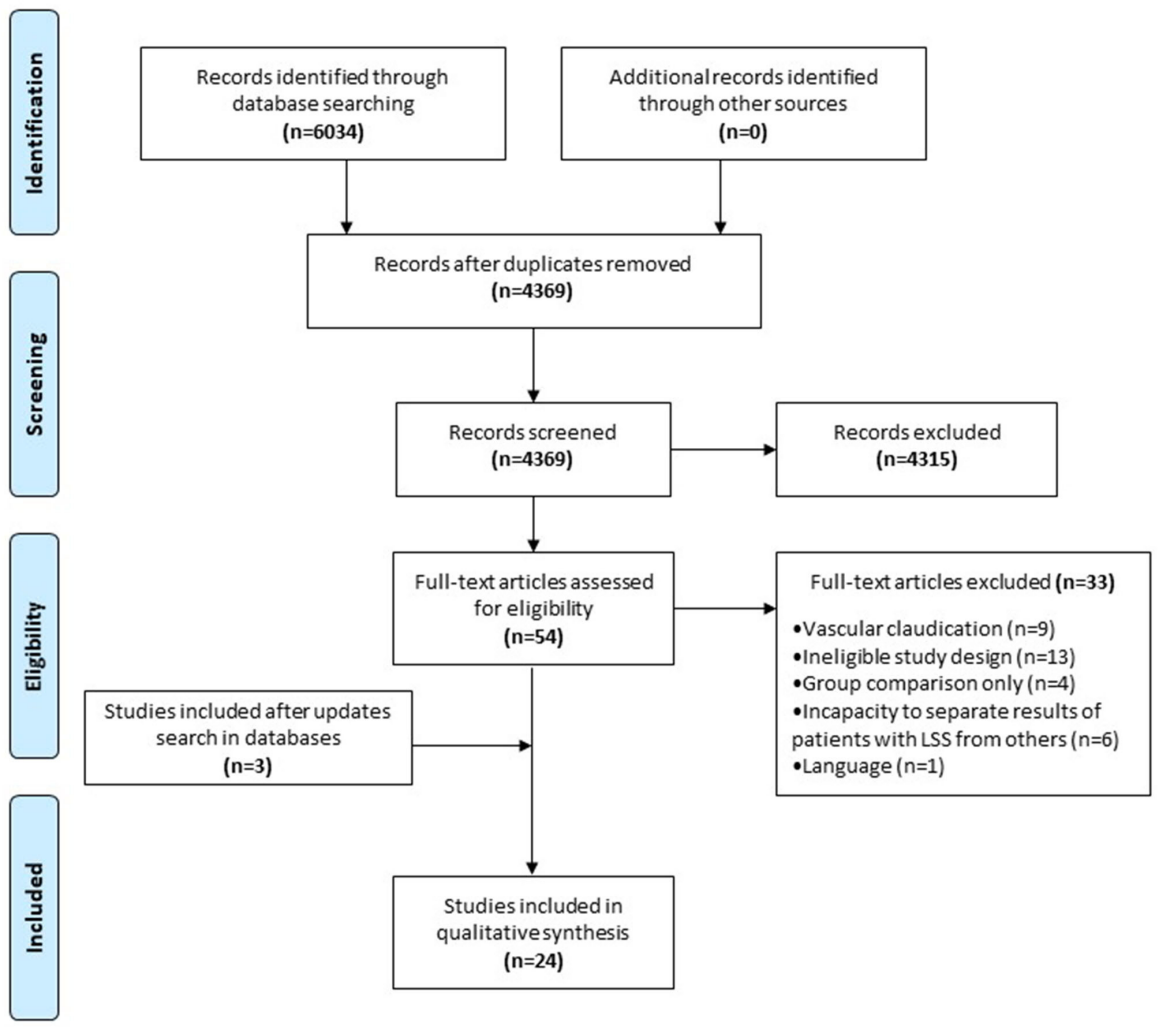
PRISMA flowchart.

The initial literature search identified 6,034 possible studies for inclusion in the scoping review. Following the removal of duplicates (*n* = 1,665), 4,369 studies remained. Initial screening of titles and abstracts resulted in the exclusion of 4315 articles that did not meet inclusion criteria. Out of the remaining 54 full-text articles, 21 fulfilled the inclusion criteria. Frequent updates of the search strategy were conducted during the review process. In light of the last update conducted on July 15, 2021, three additional articles fulfilling inclusion criteria were found bringing the final number of included studies to 24. All studies were published between 2007 and 2020 from 11 different countries over 3 continents (see Supplement Material 2 *for more details*). Figure 1 presents the study selection flowchart.

### Participants

Out of the 24 studies, seventeen were cross-sectional studies, five were prospective observational studies, one was a secondary analysis of a RCT, and one was a retrospective observational study. Sample sizes ranged from 12 to 1009 participants. In addition, out of the 2,973 participants included in the 24 studies, 1,694 had symptomatic LSS with NC. In most studies (*n* = 17), LSS was diagnosed using a combination of clinical assessment such as patient's history and/or physical examination, and findings of MRI. Regarding NC, only seven studies described NC as pain, numbness, weakness, or tingling in the lower extremity brought on by lumbar extension, standing, or walking. Based on criteria provided for inclusion of participants (or introduction section when inclusion criteria prevented the definitive identification of LSS subtypes), 974 presented with central LSS, 246 presented with combined central LSS and spondylolisthesis, 66 presented with either central or foraminal stenosis, 54 with either central or lateral stenosis, 49 with either central LSS or a combination of central and foraminal stenosis or lateral stenosis and 14 presented with lateral LSS. Finally, there were 291 patients for which the exact LSS type (e.g., central) was not explicitly described. Mean age of participants ranged from 58 to 76.9 years old across studies. Extracted data regarding population are presented in Supplement Material 2.

### Quality Assessment

The 24 studies were assessed for quality using the AXIS tool. Two studies (27, 28) scored between 6 and 10, 14 studies (15, 19, 21, 29–39) scored between 11 and 15, and 8 studies (40–47) scored between 16 and 19. The items from the AXIS tool are reported in Supplement Material 3.

### Associations

Surprisingly, no study investigated associations between gait pattern characteristics and walking capacity or functional tasks. However, a few studies reported associations between either physical or psychological factors and gait pattern characteristics. Considering that gait pattern characteristics do have a direct impact on walking capacity, these unplanned associations have been extracted and are now presented as a third category of associations along with walking capacity and functional tasks. All reported associations (significant or not) between either physical or psychological factors, and measures of walking capacity, functional tasks, or gait pattern characteristics are presented in Supplementary Table 1.

**Figure 2:**
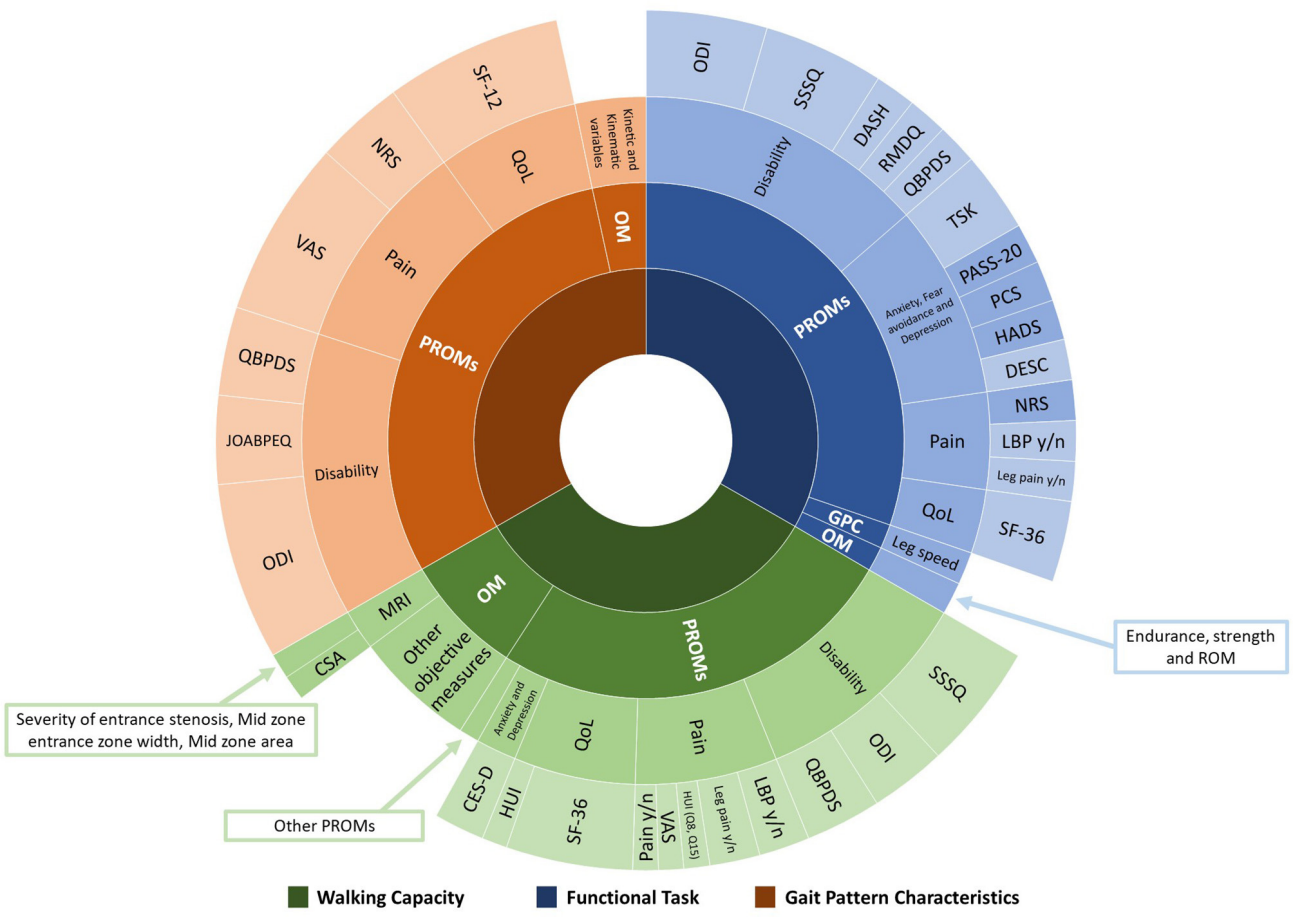
Range and proportion of tools used to assess physical and psychological factors associated with walking capacity, functional task, and gait pattern characteristics. PROMs, Patient-Reported Outcome Measures; OM, Objective Measures; GPC, Gait Pattern Characteristics; QoL, Quality of Life; MRI, Magnetic Resonance Imaging; ODI, Oswestry Disability Index; SSSQ, Swiss Spinal Stenosis Questionnaire; DASH, Disability of the Arm, Shoulder and Hand questionnaire; RMDQ, Roland-Morris Disability Questionnaire; QBPDS, Quebec Back Pain Disability Scale; TSK, Tampa Scale of Kinesiophobia; PASS-20, Pain Anxiety Symptoms Scale; PCS, Pain Catastrophizing Scale; HADS, Hospital Anxiety and Depression Scale; DESC, Rasch-based Depression Screener; NRS, Numeric Rating Scale; LBP, Low Back Pain; SF-36, 36-item Short Form Health Survey; SF-12, Medical Outcomes Short-Form 12; HUI, Health Utilities Index Mark 3; VAS, Visual Analog Scale; CES-D, Center of Epidemiological Studies Depression Scale; CSA, Cross-sectional area; JOABPEQ, Japanese Orthopedic Association Back Pain Evaluation Questionnaire.

There was significant heterogeneity within both PROMs and objective measures used to assess physical and psychological factors for each category of outcome measures among the studies. Figure 2 illustrates the range of outcome measures reported.

**Figure 3:**
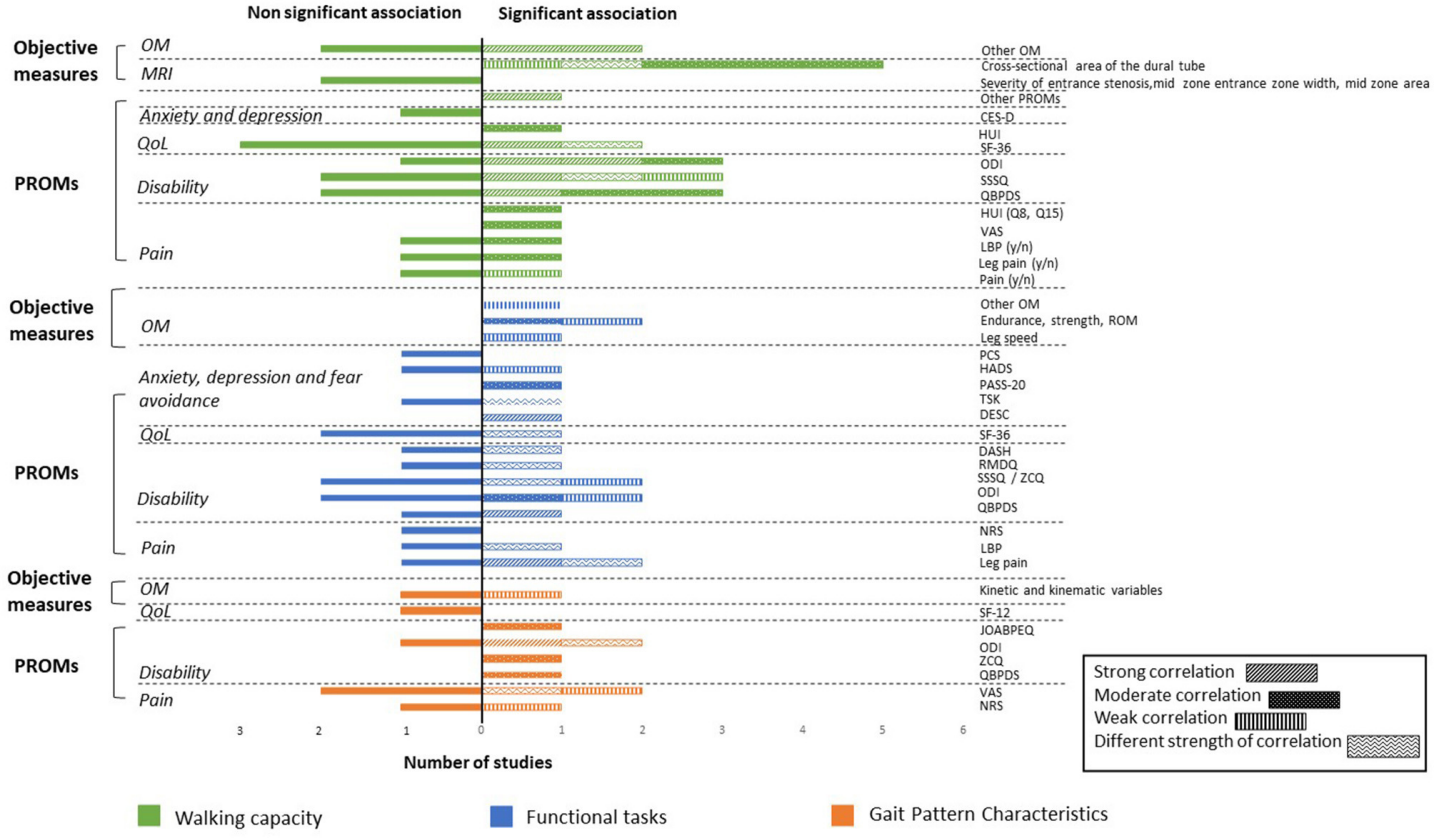
Associations between walking capacity, gait pattern characteristics, functional tasks, and PROMs and objective measures expressed in number of studies. PROMs, Patient-Reported Outcome Measures; OM, Objective Measures; QoL, Quality of Life; MRI, Magnetic Resonance Imaging; CES-D, Center of Epidemiological Studies Depression Scale; HUI, Health Utilities Index Mark 3; SF-36, 36-item Short Form Health Survey; SF-12, Medical Outcomes Short-Form 12; ODI, Oswestry Disability Index; SSSQ, Swiss Spinal Stenosis Questionnaire; ZCQ, Zurich Claudication Questionnaire; QBPDS, Quebec Back Pain Disability Scale; LBP, Low Back Pain; VAS, Visual Analog Scale; ROM, Range of Motion; PASS-20, Pain Anxiety Symptoms Scale; PCS, Pain Catastrophizing Scale; HADS, Hospital Anxiety and Depression Scale; TSK, Tampa Scale of Kinesiophobia; DESC, Rasch-based Depression Screener; DASH, Disability of the Arm, Shoulder and Hand questionnaire; RMDQ, Roland-Morris Disability Questionnaire; NRS, Numeric Rating Scale; JOABPEQ, Japanese Orthopedic Association Back Pain Evaluation Questionnaire.

As a large range of associated factors were retrieved from the 24 studies, only significant associations with walking capacity, functional tasks, and gait pattern characteristics are reported herein. Additional details regarding non-significant associations are reported in Supplement Material 4. All associations are illustrated in Figure 3.

#### Physical and Psychological Factors Associated With Walking Capacity

##### PROMs

###### Pain.

Overall, conflicting results were found based on the four studies reporting on the association between leg and/or back pain and walking capacity (distance or time). More specifically, Ishimoto et al. reported a weak positive association between symptomatic LSS (lower limb and/or buttock pain) and the 6-m walking time at maximal pace (vs. usual pace) (31). Tomkins-Lane and Battie (15) reported moderate positive correlations between walking distance and number of years with back pain and number of years with leg pain. They also reported moderate negative associations between walking capacity and intensity of leg pain before walking, and the items 8 (pain and discomfort over the past week affecting activities) and 15 (pain and discomfort over the past week requiring medication) of the Health Utilities Index Mark 3 (HUI) questionnaire (15). The other two studies did not report significant associations between pain and walking capacity (28, 40).

###### Disability.

Overall, conflicting results were found based on the six studies reporting on the association between disability and walking capacity (distance or time).

Three studies (28, 40, 44) measured disability using the Quebec Back Pain Disability Scale (QBPDS), of which only one showed a strong negative correlation between the total QBPDS score and walking distance (40). When looking at the questionnaire individual subscales, four (walk, reach, run, and groceries) were strongly and negatively correlated with total walking distance and one (stand) was strongly and negatively correlated with walking time to first symptoms.

All four studies using the Swiss Spinal Stenosis Questionnaire found moderate to strong negative correlations with walking capacity (15, 40, 41, 46). Specifically, Drury et al. reported a moderate association between walking distance and the SSSQ total score. Regarding the SSSQ individual subscales, results among the four studies were conflicting. Drury et al. reported moderate to strong negative correlations between walking capacity and the physical function and each component of the symptom subscale (pain, sensory and neuroischemic) (41). Thornes et al. reported a moderate negative correlation between walking capacity and the physical function subscale and Tomkins and Battie reported a moderate negative correlation between walking capacity and the symptom subscale. However, two studies also reported no correlation between walking capacity and some SSSQ subscales (40, 46).

All three studies using the Oswestry Disability Index (ODI) found weak to strong correlations with total walking distance (15, 40, 44). However, Conway et al. reported a non-significant correlation between the ODI and walking capacity regarding time to first symptoms (40).

###### Quality of Life.

Overall, conflicting results were found based on the five studies reporting on the association between quality of life and walking capacity (distance or time) (15, 37, 40, 41, 44).

All three studies reporting on QoL using the 36-item Short Form Health Survey (SF-36) reported at least one significant associations of varying strength with walking distance (37, 40, 41). Of these, one reported a strong positive correlation between SF-36 and walking distance (41). When looking at the questionnaire individual subscales, three studies reported moderate to strong positive correlations between the physical functioning (PF) subscale and walking distance (37, 40, 41). Moreover, Drury et al. (41) reported moderate positive correlations between four of the subscales (role physical, bodily pain, general health index and social functioning) and walking distance whereas they reported a weak positive correlation between the vitality subscale and walking distance. Out of the two studies reporting on the association between the mental health subscale and walking distance (37, 41), only one reported a moderate positive correlation (37). One study used a shorter version of the SF-36 (SF-12) and reported no significant association (44). Finally, one study reported a moderate positive correlation between health-related QoL using the HUI questionnaire and walking capacity (15).

###### Anxiety and Depression.

One study assessed depression using the CES-D and reported no significant correlation between depression status and walking distance (19).

###### Estimated Walking Distance.

One study assessed estimated walking distance and showed a strong significant correlation between this estimated and measured walking distances (40).

##### Objective Outcome Measures

###### MRI Findings.

Overall, conflicting results were found based on the three studies reporting on the association between MRI characteristics and walking capacity (distance or time) (19, 27, 33). One study reported a negative association between the cross-sectional area of the dural tube measured at L1/L2 and walking distance (19). Two studies reported no significant correlation between MRI findings and walking distance (27, 33).

###### Other Objective Outcome Measures.

Overall, conflicting results were found based on the six studies reporting on the association between other objective measures and walking capacity (15, 19, 30, 36, 40). Specifically, one study reported a strong positive correlation between maximum time of continuous activity per day over a 7-day period (at a minimum of low intensity) and walking distance (40), and another study reported a moderate positive correlation between daily step count and walking distance (35). One study showed a moderate negative correlation between balance problems and walking capacity (15). One study reported a negative association between BMI and walking distance, and a positive association between functional status (combination of tests) and walking distance (19). One study reported a strong negative correlation between trunk postural sway and maximum walking distance (36). Two of these studies also showed no significant association between other objectives measures and walking capacity (36, 40). Finally, one study reported a moderate negative correlation between handgrip strength and walking time and a weak positive correlation between handgrip strength and walking distance (30).

#### Physical and Psychological Factors Associated With Functional Tasks

##### PROMs

###### Pain.

Overall, conflicting results were found based on the four studies reporting on the association between pain outcomes and functional tasks (31, 35, 38, 40). More specifically, strong positive correlations were found between leg pain severity and overall activity per day measured with an activity monitor, as well as maximum time of continuous activity per day (40).

Pryce et al. reported moderate to strong correlations between back pain (intensity and related function), leg pain (related function), and physical activity (volume and duration). Physical activity intensity was not associated with either back or leg pain intensity and function. They also reported moderate to strong negative correlations between back or leg pain intensity and bout length or maximum bout length of meaningful activity. However, back pain intensity was negatively correlated with bout length and maximum bout length only at meaningful physical activity intensity, while leg pain intensity was only correlated at moderate intensity (between 1.5 and 2.99 METs) (38). All other associations between pain and functional tasks were non-significant (31, 35).

###### Disability.

Overall, conflicting results were found based on the five studies reporting on the association between disability and functional tasks (32, 35, 38, 40, 46, 47).

Conway et al. reported strong negative correlations between disability, measured using the run subscale of the QBPDS questionnaire, and overall activity per day, as well as between disability and time of continuous activity. All other correlations between the QBPDS and functional tasks were not significant (40).

Four studies conducted correlation analyses between the ODI and functional tasks (32, 38, 40, 47). Results from Pryce et al. (38) showed moderate to strong negative correlations between disability and physical activity volume, intensity, and duration. They also showed moderate to strong negative correlations between disability and maximum bout length at meaningful intensity and at moderate intensity during ambulatory behavior. The ODI was only correlated with bout length during ambulatory behavior at moderate intensity. Thornes et al. reported an association between stability in gait and the total ODI score. All other associations between ODI and functional tasks were not significant (32, 40).

The Roland-Morris Disability Questionnaire (RMDQ) was used in the study by Pryce et al. (38) to assess disability. In this study, authors reported moderate to strong negative correlations between disability and physical activity volume, intensity, and duration. They also reported moderate to strong negative correlations between disability and bout length and maximum bout length during ambulatory behavior (38).

Three studies reported on the correlation between the SSSQ [also known as the Zurich Claudication Questionnaire (ZCQ)] total score or subscales and functional tasks (35, 46, 47). The results of Thornes et al. (46) showed negative moderate correlations between the symptoms subscale and the 30-second Sit-to-Stand test, and the One Leg Stance test while they showed a positive moderate correlation between the symptoms subscale and the stair climbing test. The authors also reported a moderate correlation between the physical function subscale and stair climbing (46) and a weak association between the physical function subscale and the score of the Mini-BESTest (47). However, Minetama et al. (35) reported non-significant correlations between daily step count and the symptom subscale or the physical function subscale of the SSSQ.

Finally, Price et al. reported moderate to strong negative correlations between disability measured using the DASH questionnaire and physical activity volume, intensity, and duration, as well as bout length and maximum bout length during ambulatory behavior (38).

###### Quality of Life.

Overall, conflicting results were found based on the two studies reporting on the association between QoL and functional tasks (38).

Pryce et al. reported moderate to strong positive correlations between the SF-36 total score and physical activity (volume, duration, intensity), and ambulatory behavior (bout length and maximum bout length) at both meaningful and moderate intensity (38). Further details about correlations between functional task and all SF-36 subscales are presented in Supplementary Table 1.

###### Depression, Anxiety, and Fear Avoidance.

Two studies reported on the association between either depression or fear avoidance and functional tasks (21, 35). Depression was strongly and negatively correlated with the patient's participation in social, daily and work-related activities using the Aachen Activity and Participation Index (AAPI). Depression was also moderately and positively correlated with lower extremity function using the RehaCAT lower extremity subscale and with activities of daily living when using the RehaCAT lower extremity subscale. Minetama et al. (35) reported moderate negative correlations between daily step count and the total score, the cognitive anxiety subscale, the escape/avoidance subscale, and the fear subscale of the Pain Anxiety Symptom Scale (PASS-20). The authors also reported a weak negative correlation between daily step count and depression as measured by the Hospital Anxiety and Depression Scale (HADS) but no correlation with anxiety also measured by the HADS (35).

Furthermore, two studies reported on the association between kinesiophobia and functional tasks (21, 35). The somatic focus subscale of the Tampa Scale of Kinesiophobia (TSK) was strongly and positively correlated with the RehaCAT lower extremity subscale while the activity avoidance subscale was moderately and positively correlated with the RehaCAT lower extremity subscale. The score of the RehaCAT activities of daily living subscale was moderately and positively correlated with the somatic focus and the activity avoidance subscales of the TSK (21). The correlation between daily step count and kinesiophobia was not significant (35).

Finally, one study reported a correlation between daily step count and pain catastrophizing as measured with the Pain Catastrophizing Scale (PCS) (35). However, the correlations between each subscale of the PCS were not significantly correlated with daily step count.

##### Objective Outcome Measures

###### Other Objective Outcome Measures.

Two studies reported on the associations between other objective measures and functional tasks performance. Schmidt et al. (39) reported weak associations between either trunk extensor muscle endurance, leg strength asymmetry, or leg speed during a leg press and the Short Physical Performance Battery score. They also reported weak associations between either trunk extensor muscle endurance, knee flexion ROM, or knee extension strength asymmetry and the Habitual Gait Speed test, and a weak association between leg strength and the Chair Stand test (39). Finally, one study reported a moderate negative correlation between handgrip strength and walking steps (30).

#### Physical and Psychological Factors Associated With Gait Pattern Characteristics

##### PROMs

###### Pain Severity.

Overall, conflicting results were found based on the three studies reporting on the association between pain severity and gait pattern characteristics (29, 34, 43). To assess pain severity, one study used the 11-point Numeric Rating Scale (NRS) and two studies used a Visual Analog Scale (VAS) (29, 34, 43). Only back pain, measured using the NRS, was weakly and positively correlated to walking velocity (29). Regarding leg pain, Kuwahara et al. (34) reported a moderate negative correlation between leg pain and peak trunk tilt during walking (34). Other significant correlations were reported between pain severity (location unspecified) and width of base of support, the Gait Disability Index (GDI), peak lumbar tilt pre- and post-walking, and changes in pelvis tilt variation during stance (43). All other gait pattern characteristics measured in these studies were non-significant.

###### Disability.

Overall, conflicting results were found based on the three studies reporting on the association between disability and gait pattern characteristics (29, 42, 44).

Out of two studies using the ODI to assess disability (29, 44), one study reported strong positive correlations between disability and gait velocity and step length, as well as a weak positive correlation between disability and base of support (29). The same authors also reported that other gait pattern characteristics such as cadence, lumbar proprioception (except for left lateral bending) and ROM were not correlated with the ODI score. The second study reported a strong correlation between disability and free walking speed (44).

One study reported a moderate correlation between disability, measured using the QBPDS, and free walking speed (44).

Finally, one study reported weak positive associations between disability, measured using the JOABPED total score or its individual subscales, and short stride when walking (42).

###### Quality of Life.

One study reported non-significant correlations between QoL using the SF-12 and gait pattern characteristics (29).

##### Objective Outcome Measures

###### Other Objective Outcome Measures.

One study reported weak positive correlations between anterior trunk flexion angle during walking and step length as well as maximum ankle plantar flexion moment (45).

## Discussion

This systematic scoping review explored the current state of scientific knowledge regarding the associations between physical or psychological factors and walking capacity in patients with LSS. Results show that physical factors are more commonly studied than psychological factors with 22 studies reporting on physical factors and 3 reporting on psychological factors. The systematic scoping review highlighted the use of a wide range of PROMs and objective measures, with disability being the most frequently reported outcome measure followed by pain. Among objective measures, reported tools were heterogenous with most used in no more than one study. A third outcome category (gait pattern characteristics) was added following data extraction given that no study directly assessed gait pattern characteristics in relation to walking capacity. Considering the impact of gait pattern characteristics on decreased walking capacity, associations between physical or psychological factors and gait pattern characteristics were reported. Results among studies were conflicting regarding associations between PROMs and either walking capacity, functional tasks, or gait pattern characteristics. Results were also conflicting for associations between objective measures and either walking capacity, functional tasks, or gait pattern characteristics. Some outcome measures, however, were clearly associated with several measures related to walking capacity. For instance, walking capacity was significantly associated with pain outcomes, disability, estimated walking distance, and cross-sectional area of the dural tube in the lumbar spine L1/L2. Among the included studies, functional tasks were associated with physical factors such as lower back and upper limb disability, and lower limb endurance, strength, ROM, and speed. Strong significant correlations were also found between gait pattern characteristics (speed and stride) and disability outcomes. These results clearly highlight the intricate and heterogeneous presentation of symptomatic LSS.

Conflicting results can possibly be explained by the heterogeneity of walking capacity tests used across studies. Most tests were not validated in people with symptomatic LSS (28, 31–33, 36, 41, 44, 46), although a few were validated in elderly individuals (31, 32, 44). Out of nine walking tests, only four (Self-Pace walking tests, Shuttle walking test, Treadmill walking test and 6-meter walking test) were validated and/or reliable specifically for patients with LSS (see Supplement Material 5 for further details). Further studies are needed to assess the validity of commonly used walking tests in patients with symptomatic LSS. Validated walking test for patient with LSS should be used to better understand walking limitations caused by LSS.

The most commonly reported psychological factor was depression and results were conflicting. The Rasch-based Depression Screener (DESC) and PASS-20 questionnaire were associated with decreased performance in functional tasks. Other psychological factors including anxiety, depression and kinesiophobia were either not associated or showed conflicting evidence of association with walking capacity, functional tasks, or gait pattern characteristics. A comprehensive assessment of walking capacity combined with a better knowledge of the physical and psychological factors associated with walking capacity will help health care professionals identified targeted rehabilitation strategies.

The present systematic scoping review highlighted conflicting results among studies reporting on the association between PROMs or objectives measures and measures related to walking capacity (i.e., walking capacity, functional tasks, and gait pattern characteristics). The wide range of questionnaires used to assess the same outcome could also explain some of these conflicting results. For instance, disability was reported using 6 different questionnaires, with only one being specifically validated for LSS (the SSSQ). Low back pain and/or leg pain were also reported among studies using different tools (presence or absence thereof, VAS, NRS, or HUI). The most commonly reported QoL questionnaire used was the SF-36. However, its association with walking capacity and functional tasks was unclear. On the other hand, the HUI, also used to assess QoL, was associated with greater walking capacity. Objective measures were all different across studies and such heterogeneity impeded our ability to determine whether specific outcome measures are related to walking capacity in patients with LSS. In addition, no study specifically assessed gait pattern characteristics such as stride, walking phases, or walking velocity in relation to walking distance or walking time. It seems important to evaluate these characteristics in order to establish if deviations from normal gait pattern are present in patients with symptomatic LSS. Knowing that risk of fall is increased in populations with mobility impairment (32), a better understanding of walking gait characteristics that can modify walking capacity is needed to know if these walking parameters can also contribute to an increased risk of fall. As such, slower walking speed seems to be related to increased fear of falling (48). Given that some changes in gait pattern normally occur with aging, it is important to know if additional changes are brought on by symptomatic LSS.

Another possible explanation for the conflicting results is the lack of a clear definition for LSS in the studies. Diagnostic ascertainment for inclusion of participants was not clearly mentioned in some studies. Furthermore, many studies reported only the range of included LSS subtypes among participant instead of providing the exact number of individuals per subtype. They also did not clearly describe affected levels.

### Clinical Implications

For clinicians, understanding which walking parameters are modified in patients with LSS would provide new insights on the consequences brought on by LSS on gait. If any of these parameters are identified as associated factors, clinicians will be able to establish a treatment plan and monitor clinical evolution more closely over time. Clinicians are also aware that the patient's psychological state can have an impact on the prognosis of musculoskeletal disorders. For instance, patients with LSS reporting depression or at high risk of developing depression show poorer outcomes over time (49). However, more studies are needed to inform the possible implications of psychological status on walking-related functions in patients with LSS.

The Self-Paced Walking Test (SPWT) was the most used test to assessed walking capacity in patients with LSS and was the only reliable and validated one for this specific population. Results from the present systematic scoping review suggest that subjective evaluation tools such as the SSSQ and ODI were the disability-related questionnaires the most used and for which there were many significant associations with the three domains (walking capacity, functional tasks, and gait pattern characteristics). Regarding QoL, the most used questionnaire was the SF-36 but results were conflicting across studies. MRI findings (cross-sectional area) were associated with walking capacity. Regarding objective measures and psychological factors, current evidence suggests that further studies are needed to be able to better formulate recommendations for clinicians. Exploring other biomechanical walking parameters such as minimal toe clearance, speed, simple and double stance and step width can be interesting indicators to consider when assessing walking capacity in future studies.

### Strengths and Limitations

To our knowledge, this is the first systematic scoping review to assess physical and psychological factors associated with walking capacity in patients with LSS. One strength of this review is that every step was consistent with the current standards for conducting a systematic scoping review (24, 25). It is not, however, without limitation. The first limitation of our study was the low number of studies that provided a direct evaluation of walking capacity. Only studies published in English, French or Spanish were considered for this scoping review. We cannot rule-out that additional relevant evidence may have been published in other languages. Moreover, most of the studies did not specify the level and location of the LSS limiting the clinical interpretation of the results with regards to distinction of LSS subtypes.

## Conclusion

The present systematic scoping review allowed to identify physical and psychological factors that are associated with walking capacity in patients with symptomatic LSS. However, a large number of associations reported between walking capacity and physical or psychological factors were conflicting especially when correlated directly with the assessment of walking capacity.

## Data Availability Statement

The original contributions presented in the study are included in the article/Supplementary Material, further inquiries can be directed to the corresponding author/s.

## Author Contributions

MH was the principal investigator of this scoping review, participated in the study design, literature search, data extraction, results formatting, and as well as the manuscript writing. JBD participated in the data extraction and results formatting. AAM and MD participated in the study design, overall supervision of the project, and manuscript writing and revision. AAM was also involved in the screening process as the third person to obtain consensus (sorting and quality rating). All authors read and approved the final manuscript.

## Funding

The study was funded by the Chaire de recherche internationale en santé neuromusculosquelettique and its partner the Centre intégré universitaire de santé et de services sociaux de la Mauricie-et-du-Centre-du-Québec.

## Conflict of Interest

The authors declare that the research was conducted in the absence of any commercial or financial relationships that could be construed as a potential conflict of interest.

## Publisher's Note

All claims expressed in this article are solely those of the authors and do not necessarily represent those of their affiliated organizations, or those of the publisher, the editors and the reviewers. Any product that may be evaluated in this article, or claim that may be made by its manufacturer, is not guaranteed or endorsed by the publisher.

## References

[1] Ammendolia C Côté P Southerst D Schneider M Budgell B Bombardier C. Comprehensive nonsurgical treatment versus self-directed care to improve walking ability in lumbar spinal stenosis: a randomized trial. Arch Phys Med Rehabil. (2018) 99:2408–19 e2. 10.1016/j.apmr.2018.05.014 29935152

[2] Budithi S Dhawan R Cattell A Balain B Jaffray D. Only walking matters-assessment following lumbar stenosis decompression. Eur Spine J. (2017) 26:481–7. 10.1007/s00586-016-4881-x 27904964

[3] Kobayashi S. Pathophysiology, diagnosis and treatment of intermittent claudication in patients with lumbar canal stenosis. World J Orthop. (2014) 5:134–45. 10.5312/wjo.v5.i2.134 24829876 PMC4017306

[4] Hartvigsen J Hancock MJ Kongsted A Louw Q Ferreira ML Genevay S. What low back pain is and why we need to pay attention. Lancet. (2018) 391:2356–67. 10.1016/S0140-6736(18)30480-X 29573870

[5] Kalichman L Cole R Kim DH Li L Suri P Guermazi A. Spinal stenosis prevalence and association with symptoms: the Framingham Study. Spine J. (2009) 9:545–50. 10.1016/j.spinee.2009.03.005 19398386 PMC3775665

[6] Jensen RK Jensen TS Koes B Hartvigsen J. Prevalence of lumbar spinal stenosis in general and clinical populations: a systematic review and meta-analysis. Eur Spine J. (2020) 29:2143–63. 10.1007/s00586-020-06339-1 32095908

[7] Comer CM Redmond AC Bird HA Conaghan PG. Assessment and management of neurogenic claudication associated with lumbar spinal stenosis in a UK primary care musculoskeletal service: a survey of current practice among physiotherapists. BMC Musculoskelet Disord. (2009) 10:121. 10.1186/1471-2474-10-121 19796387 PMC2762954

[8] Genevay S Atlas SJ. Lumbar spinal stenosis. Best Pract Res Clin Rheumatol. (2010) 24:253–65. 10.1016/j.berh.2009.11.001 20227646 PMC2841052

[9] Singh K Samartzis D Vaccaro AR Nassr A Andersson GB Yoon ST. Congenital lumbar spinal stenosis: a prospective, control-matched, cohort radiographic analysis. Spine J. (2005) 5:615–22. 10.1016/j.spinee.2005.05.385 16291100

[10] Lurie J Tomkins-Lane C. Management of lumbar spinal stenosis. BMJ. (2016) 352:h6234. 10.1136/bmj.h6234 26727925 PMC6887476

[11] Tomkins-Lane C Melloh M Wong A. Diagnostic tests in the clinical diagnosis of lumbar spinal stenosis: consensus and results of an International Delphi Study. Europ Spine J. (2020) 29:2188–97. 10.1007/s00586-020-06481-w 32519030

[12] Tomkins-Lane CC Holz SC Yamakawa KS Phalke VV Quint DJ Miner J. Predictors of walking performance and walking capacity in people with lumbar spinal stenosis, low back pain, and asymptomatic controls. Arch Phys Med Rehabil. (2012) 93:647–53. 10.1016/j.apmr.2011.09.023 22365377 PMC3319255

[13] Williamson E Ward L Vadher K Dutton SJ Parker B Petrou S. Better Outcomes for Older people with Spinal Trouble (BOOST) trial: a randomised controlled trial of a combined physical and psychological intervention for older adults with neurogenic claudication, a protocol. BMJ Open. (2018) 8:e022205. 10.1136/bmjopen-2018-022205 30341124 PMC6196848

[14] Barz T Melloh M Staub L Roeder C Lange J Smiszek F-G. The diagnostic value of a treadmill test in predicting lumbar spinal stenosis. Europ Spine J. (2008) 17:686–90. 10.1007/s00586-008-0593-1 18259784 PMC2367408

[15] Tomkins-Lane CC Battie MC. Predictors of objectively measured walking capacity in people with degenerative lumbar spinal stenosis. J Back Musculoskelet Rehabil. (2013) 26:345–52. 10.3233/BMR-130390 23948821

[16] Ammendolia C Côté P Rampersaud YR Southerst D Budgell B Bombardier C. The boot camp program for lumbar spinal stenosis: a protocol for a randomized controlled trial. Chiropr Man Therap. (2016) 24:25. 10.1186/s12998-016-0106-y 27433335 PMC4948101

[17] Marchand AA Suitner M O'Shaughnessy J Chatillon CE Cantin V Descarreaux M. Feasibility of conducting an active exercise prehabilitation program in patients awaiting spinal stenosis surgery: a randomized pilot study. Sci Rep. (2019) 9:12257. 10.1038/s41598-019-48736-7 31439877 PMC6706402

[18] Rainville J Childs LA Peña EB Suri P Limke JC Jouve C. Quantification of walking ability in subjects with neurogenic claudication from lumbar spinal stenosis–a comparative study. Spine J. (2012) 12:101–9. 10.1016/j.spinee.2011.12.006 22209240 PMC3315838

[19] Zeifang F Schiltenwolf M Abel R Moradi B. Gait analysis does not correlate with clinical and MR imaging parameters in patients with symptomatic lumbar spinal stenosis. BMC Musculoskelet Disord. (2008) 9:89. 10.1186/1471-2474-9-89 18570636 PMC2441626

[20] Takenaka H Sugiura H Kamiya M Nishihama K Ito A Suzuki J. Predictors of walking ability after surgery for lumbar spinal canal stenosis: a prospective study. Spine J. (2019) 19:1824–31. 10.1016/j.spinee.2019.07.002 31302266

[21] Quack V Boecker M Mueller C Mainz V Geiger M Heinemann A. Psychological factors outmatched morphological markers in predicting limitations in activities of daily living and participation in patients with lumbar stenosis. BMC Musculoskelet Disord. (2019) 20:1–9. 10.1186/s12891-019-2918-0 31759398 PMC6875026

[22] Mullen SP McAuley E Satariano WA Kealey M Prohaska TR. Physical activity and functional limitations in older adults: the influence of self-efficacy and functional performance. J Gerontol B Psychol Sci Soc Sci. (2012) 67:354–61. 10.1093/geronb/gbs036 22473023 PMC3410698

[23] Ammendolia C Schneider M Williams K Zickmund S Hamm M Stuber K. The physical and psychological impact of neurogenic claudication: the patients' perspectives. J Can Chiropr Assoc. (2017) 61:18–31. 28413220 PMC5381486

[24] Peters MD Godfrey CM Khalil H McInerney P Parker D Soares CB. Guidance for conducting systematic scoping reviews. Int J Evidence-Based Healthcare. (2015) 13:141–6. 10.1097/XEB.0000000000000050 26134548

[25] Levac D Colquhoun H O'Brien KK. Scoping studies: advancing the methodology. Implement Sci. (2010) 5:69. 10.1186/1748-5908-5-69 20854677 PMC2954944

[26] Downes MJ Brennan ML Williams HC Dean RS. Development of a critical appraisal tool to assess the quality of cross-sectional studies (AXIS). BMJ Open. (2016) 6:e011458. 10.1136/bmjopen-2016-011458 27932337 PMC5168618

[27] Sigmundsson FG Kang XP Jönsson B Strömqvist B. Correlation between disability and MRI findings in lumbar spinal stenosis: a prospective study of 109 patients operated on by decompression. Acta Orthopaed. (2011) 82:204–10. 10.3109/17453674.2011.566150 21434811 PMC3235292

[28] Tong HC Haig AJ Geisser ME Yamakawa KS Miner JA. Comparing pain severity and functional status of older adults without spinal symptoms, with lumbar spinal stenosis, and with axial low back pain. Gerontology. (2007) 53:111–5. 10.1159/000096861 17095872

[29] Conrad BP Shokat MS Abbasi AZ Vincent HK Seay A Kennedy DJ. Associations of self-report measures with gait, range of motion and proprioception in patients with lumbar spinal stenosis. Gait Posture. (2013) 38:987–92. 10.1016/j.gaitpost.2013.05.010 23810090

[30] Inoue H Watanabe H Okami H Shiraishi Y Kimura A Takeshita K. Handgrip strength correlates with walking in lumbar spinal stenosis. Eur Spine J. (2020) 29:2198–204. 10.1007/s00586-020-06525-1 32651633

[31] Ishimoto Y Yoshimura N Muraki S Yamada H Nagata K Hashizume H. Prevalence of symptomatic lumbar spinal stenosis and its association with physical performance in a population-based cohort in Japan: the Wakayama Spine Study. Osteoarthritis Cartilage. (2012) 20:1103–8. 10.1016/j.joca.2012.06.018 22796511

[32] Kim H-J Chun H-J Han C-D Moon S-H Kang K-T Kim H-S. The risk assessment of a fall in patients with lumbar spinal stenosis. Spine. (2011) 36:E588–E92. 10.1097/BRS.0b013e3181f92d8e 21242866

[33] Kuittinen P Sipola P Aalto TJ Määttä S Parviainen A Saari T. Correlation of lateral stenosis in MRI with symptoms, walking capacity and EMG findings in patients with surgically confirmed lateral lumbar spinal canal stenosis. BMC Musculoskelet Disord. (2014) 15:247. 10.1186/1471-2474-15-247 25051886 PMC4112604

[34] Kuwahara W Kurumadani H Tanaka N Nakanishi K Nakamura H Ishii Y. Correlation between spinal and pelvic movements during gait and aggravation of low back pain by gait loading in lumbar spinal stenosis patients. J Orthopaed Sci. (2019) 24:207–13. 10.1016/j.jos.2018.09.002 30322623

[35] Minetama M Kawakami M Teraguchi M Kagotani R Mera Y Sumiya T. Associations between psychological factors and daily step count in patients with lumbar spinal stenosis. Physiother Theory Pract. (2020). 10.1080/09593985.2020.1855685. [Epub ahead of print]. 33267720

[36] Nagai K Aoyama T Yamada M Izeki M Fujibayashi S Takemoto M. Quantification of changes in gait characteristics associated with intermittent claudication in patients with lumbar spinal stenosis. J Spinal Disord Tech. (2014) 27:E136–42. 10.1097/BSD.0b013e3182a2656b 24869987

[37] Özdemir E Paker N Bugdayci D Tekdos DD. Quality of life and related factors in degenerative lumbar spinal stenosis: a controlled study. J Back Musculoskelet Rehabil. (2015) 28:749–53. 10.3233/BMR-140578 25547233

[38] Pryce R Johnson M Goytan M Passmore S Berrington N Kriellaars D. Relationship between ambulatory performance and self-rated disability in patients with lumbar spinal stenosis. Spine. (2012) 37:1316–23. 10.1097/BRS.0b013e31824a8314 22261635

[39] Schmidt CT Ward RE Suri P Kiely DK Ni P Anderson DE. Association of neuromuscular attributes with performance-based mobility among community-dwelling older adults with symptomatic lumbar spinal stenosis. Arch Phys Med Rehabil. (2017) 98:1400–6. 10.1016/j.apmr.2017.02.028 28377110 PMC5501102

[40] Conway J Tomkins CC Haig AJ. Walking assessment in people with lumbar spinal stenosis: capacity, performance, and self-report measures. Spine J. (2011) 11:816–23. 10.1016/j.spinee.2010.10.019 21145292 PMC3136653

[41] Drury T Ames SE Costi K Beynnon B Hall J. Degenerative spondylolisthesis in patients with neurogenic claudication effects functional performance and self-reported quality of life. Spine. (2009) 34:2812–7. 10.1097/BRS.0b013e3181b4836e 19940740

[42] Fujita N Sakurai A Miyamoto A Michikawa T Otaka Y Suzuki S. Stride length of elderly patients with lumbar spinal stenosis: multi-center study using the Two-Step test. J Orthopaed Sci. (2019) 24:787–92. 10.1016/j.jos.2019.01.006 30737067

[43] Garbelotti SA Jr Lucareli PRG Ramalho A Jr de Godoy W Bernal M Greve JMDA. An investigation of the value of tridimensional kinematic analysis in functional diagnosis of lumbar spinal stenosis. Gait Posture. (2014) 40:150–3. 10.1016/j.gaitpost.2014.03.013 24755459

[44] Grelat M Gouteron A Casillas J-M Orliac B Beaurain J Fournel I. Walking speed as an alternative measure of functional status in patients with lumbar spinal stenosis. World Neurosurg. (2019) 122:e591–e7. 10.1016/j.wneu.2018.10.109 31108075

[45] Igawa T Katsuhira J Hosaka A Uchikoshi K Ishihara S Matsudaira K. Kinetic and kinematic variables affecting trunk flexion during level walking in patients with lumbar spinal stenosis. PLoS ONE. (2018) 13:e0197228. 10.1371/journal.pone.0197228 29746537 PMC5944950

[46] Thornes E Robinson HS Vøllestad NK. Degenerative lumbar spinal stenosis and physical functioning: an exploration of associations between self-reported measures and physical performance tests. Disabil Rehabil. (2018) 40:232–7. 10.1080/09638288.2016.1250123 27846739

[47] Thornes E Robinson HS Vøllestad NK. Dynamic balance in patients with degenerative lumbar spinal stenosis; a cross-sectional study. BMC Musculoskelet Disord. (2018) 19:192. 10.1186/s12891-018-2111-x 29902972 PMC6003037

[48] Chamberlin ME Fulwider BD Sanders SL Medeiros JM. Does fear of falling influence spatial and temporal gait parameters in elderly persons beyond changes associated with normal aging? J Gerontol A Biol Sci Med Sci. (2005) 60:1163–7. 10.1093/gerona/60.9.1163 16183957

[49] Hébert JJ Abraham E Wedderkopp N Bigney E Richardson E Darling M. Preoperative factors predict postoperative trajectories of pain and disability following surgery for degenerative lumbar spinal stenosis. Spine. (2020) 45:E1421–30. 10.1097/BRS.0000000000003587 32541610 PMC7547903

