# Supplementary material for: Physical and Psychological Factors Associated With Walking Capacity in Patients With Lumbar Spinal Stenosis With Neurogenic Claudication: A Systematic Scoping Review
Source: Front Neurol. 2021 Sep 9;12:720662. doi: 10.3389/fneur.2021.720662 (PMC8459720; doi:10.3389/fneur.2021.720662)
Supplement: Supplementary file 1 [file Data_Sheet_1.docx]

**Supplement 1: Example of search strategy**

Database: **MEDLINE**

|  | **Non-MeSH** | **MeSH** |
| --- | --- | --- |
| **Population** | Lumbar spinal stenosis | Spinal stenosis |
|  | Neurogenic claudication | Intermittent claudication |
| **Intervention** | Walking pattern | Walking |
|  | Gait pattern | Gait analysis |
|  | Walking velocity | Walking speed |
|  | Walking speed | Walking speed |
|  | Gait velocity | Gait velocity |
|  | Gait speed | Gait velocity |
|  | Walking time | Walking |
|  | Gait duration | Gait analysis |
|  | Stride length | N/A |
|  | Step length | N/A |
|  | Spatiotemporal parameters | Spatio-temporal analysis |
|  | Step width | N/A |
|  | Walking asymmetry | Mobility limitation |
|  | Walking symmetry | Walking |
|  | Gait asymmetry | Gait analysis |
|  | Gait cycle | Gait analysis |
|  | Gait cycle duration | Gait analysis |
|  | Gait balance | Postural balance |
|  | Gait disorders | Gait disorders |
|  | Gait stability | Gait |
|  | Stair climbing | Stair climbing |
|  | Climbing | Stair climbing |
|  | Step | Exercise test |
|  | Step ascent | Exercise test |
|  | Step descent | Exercise test |
|  | Running pattern | Running |
|  | Stance phase | N/A |
|  | Swing phase | N/A |
|  | Cadence | N/A |
| **Outcomes** | Walking capacity | Walking |
|  | Walking time | Time |
|  | Walking distance | Walking |
|  | Symptoms | Signs and Symptoms |
|  | Pain | Pain |
|  | Functional capacity | N/A |
|  | Walking function | Walking |
|  | Lower limb pain | Pain |
|  | Kinesiophobia | N/A |
|  | Anxiety | Anxiety |
|  | Depression | Depression |
|  | Frailty | Frailty |
|  | Self-efficacy | Self-efficacy |

Non-MeSH results

|  | **Key words** | **Results** |
| --- | --- | --- |
| **S1** | **''lumbar spinal stenosis'' OR ''neurogenic claudication''** | 5,743 |
| **S2** | **walk* pattern* OR Gait pattern* OR run* pattern* OR walk* velocity OR walk* speed OR gait velocity OR gait speed OR run* velocity OR run* speed** | 25,620 |
| **S3** | **walk* time OR gait duration OR gait cycle duration OR gait cycle OR stance phase OR swing phase OR cadence** | 16,404 |
| **S4** | **stride length OR step length OR step width OR ''spatiotemporal parameters''** | 7,818 |
| **S5** | **''walk* asymmetr*'' OR ''walk* symmetr*'' OR ''gait asymmetr*'' OR ''gait symmetr*'' OR gait balance OR gait disorder* OR gait stability** | 12,896 |
| **S6** | **Stair* climb* OR Climb* OR step* OR step ascent OR step descent** | 711,761 |
| **S7** | **''walking capacity'' OR ''walking time'' OR ''walking distance'' OR symptoms OR pain OR functional capacit* OR ''walking function'' OR lower limb pain** | 1,556,756 |
| **S8** | **self-efficacy OR frailty OR depression OR anxiety OR kinesiophobia** | 570,333 |
| **S9** | **S2 OR S3 OR S4 OR S5 OR S6** | 750,159 |
| **S10** | **S7 OR S8** | 1,965,506 |
| **S11** | **S1 AND S9 AND S10** | 150 |

MeSH results

|  | **Key words** | **Results** |
| --- | --- | --- |
| **S1** | **''spinal stenosis'' OR ''intermittent claudication''** | 17,834 |
| **S2** | **walk* OR run* OR walk * speed OR gait velocity**  **OR Gait OR Gait disorder* OR gait analysis OR spatio-temporal analysis OR mobility limitation OR postural balance OR Stair climb* OR Exercise* test*** | 513,674 |
| **S3** | **walk* OR time OR signs symptoms OR pain** | 5,060,536 |
| **S4** | **anxiety OR depression OR frailty OR self-efficacy** | 569,901 |
| **S5** | **S3 OR S4** | 5,477,578 |
| **S6** | **S1 AND S2 AND S5** | 2,738 |
