# Supplementary material for: Physical and Psychological Factors Associated With Walking Capacity in Patients With Lumbar Spinal Stenosis With Neurogenic Claudication: A Systematic Scoping Review
Source: Front Neurol. 2021 Sep 9;12:720662. doi: 10.3389/fneur.2021.720662 (PMC8459720; doi:10.3389/fneur.2021.720662)
Supplement: Supplementary file 2 [file Data_Sheet_2.docx]

**Data extraction – Scoping review**

| **#** | **First author, year** | **Title** | **Country** | **Study Design** | **(N = )** | **Definitions** | **Population** | **Independant variables** | **Dependant variables** | **Results** | **Comments** |
| --- | --- | --- | --- | --- | --- | --- | --- | --- | --- | --- | --- |
| **1** | **Conrad et al. 2013** | Associations of self-report measures with gait, range of motion and proprioception in patients with lumbar spinal stenosis | USA | Cross-sectional | 25 | **LSS:** Spinal stenosis is defined as neurogenic claudication due to narrowing of the spinal canal lumen diameter.  Inclusion criteria for LSS = NA  **NC:** NA | Patients with symptomatic LSS (N=25)  M: F ratio = 11: 14  Age = 62 ± 14 yo | Walking velocity, stride length, cadence, base of support | ODI,  SF-12,  NRS | ***ODI:***  Strongly correlated with step length (r^2^=0.58; p<0.001) and gait velocity (r^2^=0.506; p=0.001).  Weakly correlated with the base of support (r^2^=0.363; p=0.008) and left lateral bending proprioception (r^2^=0.213; p=0.036).  There was no significant correlation between ODI and cadence (r^2^=0.182), ROM (r^2^= [0.024; 0.163]) and proprioception (extension, flexion and right lateral bending) (r^2^= [-0.053; -0.039]).  ***SF-12:***  There was no significant correlation between SF-12 and biomechanical measurements (r^2^ = [0.001 - 0.183]).  ***NRS:***  Weakly correlated with velocity (r^2^=0.189; p=0.049) and base of support (r^2^=0.219; p=0.032).  There was no significant correlation between NRS and step length, cadence, ROM and proprioception (r^2^= [0.000 - 0.177]) |  |
| **2** | **Conway et al. 2011** | Walking assessment in people with lumbar spinal stenosis: capacity, performance, and self-report measures | USA and Canada | Prospective laboratory and clinical observational study | 12 | **LSS:**  Characterized by a narrowing of the lumbar spinal canal or nerve root foramen. Symptoms, including back pain, neurological deficits, and pain  in the lower limbs.  Diagnosis was based on patient history, physical examination and MRI  **NC:** Pain, numbness, weakness, or tingling in the lower extremity brought on by lumbar extension, standing, or walking. | Patients with LSS with NC (N=12)  M: F ratio = 9: 3  Age= 66.3 ±9.8 yo | SPWT distance, SPWT distance to first symptoms, activity count/d, maximum time of continuous activity, QBPDS, ODI, SSSQ, SF-36, Walking, walking capacity, estimated walking, leg pain. | **Walking capacity** (SPWT distance and SPWT distance to first symptoms)  **Walking performance** (Activity count/d and maximum time of continuous activity) | ***Walking capacity:***  Strong correlation between SPWT distance and maximum time of continuous activity per day (r=0.63; p<0.05), QBPDS (total: r=-0.638; p<0.05, walk: r=-0.755; p<0.01, reach: r=-0.650; p<0.05, run: r=-0.664; p<0.05, groceries: r=-0.727; p<0.05), SSSQ (PF: r=-0.610; p<0.05, PF walk: r=-0.715; p<0.05, SS weak: r=-0.742; p<0.01, SS balance: r=-0.673; p<0.05), ODI (r=-0.595; p<0.05), SF-36 physical functioning (r=0.825; p<0.01) and estimated walking distance (r=0.886; p<0.01).  There was no significant correlation between SPWT distance and SPWT time to first symptoms, activity count/d, leg pain and QBPDS stand (r= [-0.551 – 0.563]).  Strong relationship between SPWT distance to first symptoms and estimated walking (r=0.659; p<0.05), QBPDS stand (r=-0.681; p<0.05) and SSSQ (PF walk: r=-0.622; p<0.05, balance: r=-0.646; p<0.05)  There was no significant correlation between SPWT distance to first symptoms and activity count/d, maximum time of continuous, total QBPDS score, ODI, SSSQ PF, SF-36 PF, walking capacity, leg pain, QBPDS (walk, reach, run and groceries) and SSSQ (SS weak) (r= [-0.628 – 0.422]).  ***Walking performance:***  Strong relationship between Activity count/d and maximum time of continuous activity (r=0.879; p<0.01), leg pain (r=0.623; p<0.05) and QBPDS run (r=-0.884; p<0.01).  There was no significant correlation between Activity count/d and QBPDS (total score, stand, walk, reach and groceries), ODI, SSSQ (PF, PF walk, SS weak and SS balance), SF-36, walking capacity and estimated walking (r= [-0.291 – 0.521]).  Strong relationship between maximum time of continuous activity and SPWT distance (r=0.629; p<0.05), activity count/d (r=0.876; p<0.01), leg pain (r=0.754; p<0.01) and QBPDS run (r=-0.825; p<0.01)  There was no significant correlation between maximum time of continuous activity and QBPDS (total, stand, walk, reach and groceries), ODI, SSSQ (PF, PF walk, SS weak and SS balance), SF-36, walking capacity and estimated walking (r= [-0.375 – 0.541]). | 13 out of 15 measures (87%) were more highly correlated to the SPWT than the activity monitor. |
| **3** | **Drury et al. 2009** | Degenerative Spondylolisthesis in Patients With Neurogenic Claudication Effects Functional Performance and Self-Reported Quality of Life | USA | Prospective study | 77 | **LSS:** NA  Diagnosis was based on clinical symptoms.  **NC:**  Characterized by pain radiating down the lower extremities in a radicular distribution when a patient is standing of walking and generally relieved by sitting down or leaning forward.  Secondary to LSS. | Patients with NC secondary to LSS  (N=77)  M: F ratio = 39: 38  LSS without spondylolisthesis (N=32)  Age = 67.1 ± 9.8 yo  LSS with fixed spondylolisthesis (n=22)  Age = 70.4 ± 10.9 yo  LSS with mobile spondylolisthesis (n=23)  Age = 68.6 ± 10.8 yo | SSSQ, SF-36 | **Walking distance** (Shuttle Walking Test) | Strong correlation between walking distance and SSSQ PF (r= -0.52; p<0.001) and SF-36 physical functioning (r= 0.60; p<0.001).  Moderate correlation between walking distance and SSSQ (total score: r=-0.50; p<0.001, pain: r= -0.27; p=0.019,sensory: r=-0.36; p=0.002, neuroischemic: r= -0.30; p=0.009), SF-36 (role physical: r= 0.35; p=0.003, bodily pain: r=0.39; p=0.001, general health index: r=0.26; p=0.033 and social functioning: r=0.31; p=0.009).  Weak correlation between walking distance and SF-36 vitality (r= 0.24; p=0.041).  There was no significant correlation between walking distance and SF-36 role emotional and mental health index subscales (r= [0.06 - 0.075]). | Mean age for all patients was not available.  Mean age was available only for subgroup of patients with LSS. However, correlations were done combining all patients from the 3 subgroups. |
| **4** | **Fujita et al. 2019** | Stride length of elderly patients with lumbar spinal stenosis: Multi-center study using the Two-Step test | Japan | Cross-sectional study | 357 | **LSS:**  Lumbar spinal canal stenosis (LSS) is a degenerative musculoskeletal disorder characterized by narrowing of the lumbar spinal  canal.  Diagnosis was based on clinical symptoms and MRI.  **NC:** NA | Patients who planned to undergo primary surgery for LSS with leg pain/NC (n=357)  M:F ratio=  201: 156  Age= 73.3 ±5.47 yo | Two-Step test  Age, sex, JOABPEQ, ZCQ, motor deficit, sagittal vertical axis, and lumbar lordosis | TUGT  Stride | Strong correlation between the Two-Step test and the TUGT (r=-0.65; p<0.001).  Univariable analysis showed a significant association between short stride with age ≥ 80 years (OR=2.8), score of lumbar function in JOABPEQ <60 (OR=2.9), score of walking ability in JOABPEQ <30 (OR=1.6), score of social life in JOABPEQ <45 (OR=1.6), score of psychological disorder in JOABPEQ <45 (OR=1.9), symptom severity in ZCQ ≥4 (OR=1.8), physical function in ZCQ ≥2.5 (OR=2.0), motor deficit (OR=3.1), and sagittal vertical axis of ≥50 mm (OR=2.4)  When adjusting all variables, associations with age ≥ 80 years (OR= 2.3), the score of lumbar function in JOABPEQ of <60 (OR=2.7), motor deficit (OR=2.7) and sagittal vertical axis of ≥50 mm (OR=2.1). |  |
| **5** | **Gaberlotti et al. 2014** | An investigation of the value of tridimensional kinematic analysis in functional diagnosis of lumbar spinal stenosis | Brazil | Cross-sectional study, uncontrolled intervention | 14 | **LSS:** Spine canal space restricted, there may be symptoms, such as pain, numbness, weakness, and  neurogenic claudication, which can worsen with effort and  improve with rest.  Diagnosis was based on medical assessment and MRI.  **NC:**  Neurogenic  claudication is related to compression of the nerve roots in the  spinal canal. | Patients with LSS (N=14)  M:F ratio= 10: 4  Age= 74.5 ±9.8 yo | GDI,  Kinematics parameters (speed, cadence, stride length, single stance, double stance, swing and trunk tilt) | Pain perception (VAS), cross-sectional area of the spinal canal | There was no significant correlation between pain perception pre-effort and kinematic parameters (r= [-0.45; 0.46]).  Strong correlation between post-effort pain perception and GDI (left side: r=-0.64; p<0.05 and right side: r=-0.53; p<0.05) but there was no other significant correlation with kinematic parameters (r= [-0.42 - 0.19]).  There was no significant correlation between cross-sectional area and GDI and kinematic parameters for both pre- and post-effort (r= [-0.34 - 0.34]). |  |
| **6** | **Grelat et al. 2019** | Walking Speed as an Alternative Measure of Functional Status in Patients with Lumbar Spinal Stenosis | France | Cross-sectional | 38 | **LSS:** NA  Diagnosis was confirmed with MRI.  **NC:** NA | Patients with LSS referred to the neurosurgery department and with the ability to walk 10m or more without help (N=38)  M: F ratio= 23: 15  Age= 69.3 ±7.9 yo | ODI, QBPDS, SF-12, estimated walking perimeter | Measured walking perimeter,  free walking speed, estimated error | Weak correlation between measured walking perimeter and ODI (r= -0.44; p= 0.005).  ***Patients estimating their walking perimeter as lower than 500m (n=21):***  Strong correlation between estimated walking perimeter and estimated error (r=-0.63; p=0.002)  ***In patients walking less than 500m (n=7):***  Strong correlation between the measured free walking speed and ODI (r= -0.71; p=0.001) and QBPDS (r= -0.5; p= 0.0008)  There was no association between SF-12 and estimated walking perimeter, measured walking perimeter and error estimation. |  |
| **7** | **Igawa et al. 2018** | Kinetic and kinematic variables affecting trunk flexion during level walking in patients with lumbar spinal stenos | Japan | Cross-sectional | 111 | **LSS:** Caused by narrowing of the spinal canal or the various tunnels through which nerves and other structures communicate with that canal.  Diagnosis was based on clinical symptoms and MRI.  **NC:** Pain in legs with walking, which is caused by blockage of blood outflow from around the nerves. | Patients with acquired degenerative LSS with leg pain (N=111)  M: F ratio= 65: 51  Age= 70.9 ±6.2 yo | Back pain, JOABPEQ, VAS, Step length, velocity, one gait cycle time, maximum pelvis anterior tilt angle, maximum hip flexion and extension angle and moment, maximum knee flexion and extension angle and moment, maximum ankle dorsi and plantar flexion angle and moment, maximum and minimum hip, knee and ankle power | Anterior trunk flexion angle | Moderate correlation between anterior trunk flexion angle and maximum pelvis anterior tilt (r=0.29; p<0.01), maximum hip flexion angle (r=0.27; p<0.01), maximum hip extension angle (r=0.47; p<0.01), maximum knee flexion angle (r=0.33; p<0.01), maximum knee extension angle (r=0.33; p<0.01), maximum hip flexion moment (r=0.44; p<0.01) and minimum hip power (r=-0.38; p<0.01).  Weak correlation between anterior trunk flexion angle and step length (r=0.17; p=0.01), maximum ankle plantar flexion moment (r=0.20; p<0.01) and maximum knee power (r=0.21; p<0.01)  There was no significant correlation between max trunk flexion angle and VAS, JOABPEQ, velocity, gait cycle time, maximum pelvis rotation angle, maximum ankle dorsi flexion angle, maximum ankle plantar flexion angle, maximum hip extension moment, maximum knee flexion moment, maximum knee extension moment, maximum ankle dorsi flexion moment, maximum hip power, minimum knee power, maximum ankle power and minimum ankle power (r=[-0.11 - 0.12]).  Maximum hip extension angle (β=0.42), maximum hip flexion moment (β=-0.35) and step length (β=0.26) were able to explain 29% of the variance. |  |
| **8** | **Inoue et al. 2020** | Handgrip strength correlated with walking in lumbar spinal stenosis | Japan | Retrospective | 183 | **LSS:** Symptoms of LSS involve lower back pain, lower extremity pain, lower extremity numbness, lower muscle weakness and gait disturbance.  Inclusion criteria for patients with LSS: confirmed diagnosis through neurologic examination and imaging.  **NC:** NA | Patients with LSS NC or radicular leg pain  M:F ratio = 128: 55  Age = 70.5 yo (range 36-88 yo) | 10-meter walk test (time and step), 300m walking distance test, psoas muscle index, leg extension power, intermittent claudication | Handgrip strength | Strong correlation between handgrip strength and leg power extension (r=0.723 ; p<0.001)  Weak significant negative correlation between handgrip strength and 10-meter walk test time (r=-0.269 ; p<0.001), and 10-meter walk test steps (r=-0.352 ; p<0.001). There was a significant weakly positive correlation between handgrip strength and intermittent claudication (measure with the 300 m walking distance test) (r=0.201 ; p=0.008). |  |
| **9** | **Ishimoto et al. 2012** | Prevalence of symptomatic lumbar spinal stenosis and its association with  physical performance in a population-based cohort in Japan: the Wakayama Spine  Study | Japan | Cross-sectional study | 1009 | **LSS:** Symptomatic lumbar spinal stenosis (LSS) is usually associated  with impaired walking and other disabilities in the elderly.  Inclusion criteria for patients with LSS: NA  **NC:** NA | Total study  M: F ratio = 335: 674  Age = 66.3 ±13.6 yo  *Group 1:* symptomatic LSS (N= 94)  *Group 2:* non-symptomatic LSS  (N=915) | Symptomatic LSS | 6-m walking time at a usual pace,  6-m walking time at a maximal pace,  Chair standing time,  One leg standing time | 6-m walking time at a maximal pace was significantly associated with symptomatic LSS (OR = 1.17).  There was no significant association between 6-m walking time at a usual pace (OR=1.04), chair standing time (OR= 1.03) and one-leg standing time (OR =1.00) in symptomatic LSS. | The article did not mention the mean age for the LSS group.  The article did not mention sex ratio for the LSS group. |
| **10** | **Kim et al. 2011** | The Risk Assessment of a Fall in Patients With Lumbar Spinal Stenosis | Korea | Prospective case-control study (observational study) | 80 | **LSS:** Degenerative arthritic disease in the spine that results in decreasing function, impaired  balance, and gait deficit, with increased levels of leg and back pain.  Diagnosis was based on walking intolerance and MRI.  **NC:**  Walking impairment is the main symptom of LSS and  is caused by neurogenic claudication, which leads to limited  ambulation, decreased daily activity, and muscle weakness. | Patients with LSS with NC  (N=40)  M: F ratio= 11: 29  Age= 62.82 ±7.29 yo | ODI | Alternative-Step test, Six-Meter-Walk test, Sit-to-Stand test, TUGT | There was no significant correlation between the ODI score and the four functional mobility tests. |  |
| **11** | **Kuittinen et al. 2014** | Correlation of lateral stenosis in MRI with symptoms, walking capacity and EMG findings in patients with surgically confirmed lateral lumbar spinal canal stenosis | Finland | Prospective study | 14 | **LSS:** Buttock or lower extremity pain, which may occur with or without back pain, associated with diminished space available for the neural and vascular elements in the lumbar spine.  Diagnosis was based on clinical and imaging findings.  **NC:** NA | Patients with LSS with leg pain and/or NC (N=14)  M:F ratio= 6: 8  Age= 58 (range 48-76) years old | MRI findings | BDI, VAS, ODI, EMG, walking distance | MRI findings (severity of entrance stenosis, mid zone entrance zone width, mid zone area) did not correlate with ODI, VAS, specific low back pain, specific leg pain, BDI and walking distance.  Abnormal EMG is significantly correlated to MRI findings (severity of mid zone stenosis) in supine position (p=0.015). | No correlation results are available in this study. Authors just mentioned if there were correlations or not. |
| **12** | **Kuwahara et al. 2019** | Correlation between spinal and pelvic movements during gait and aggravation of low back pain by gait loading in lumbar spinal stenosis | Japan | Cross-sectional | 29 | **LSS:** Lumbar spinal stenosis patients frequently complain of  numbness and pain in the lower limbs, and neurogenic intermittent  claudication.  Diagnosis was base on patient history, physical examination and MRI.  **NC:** NA | Patients with LSS with leg pain and/or NC (N= 29)  M: F ratio=  17: 12  *Group1* (n=8): leg pain and LBP changes  Age= 72 ± 7 yo  *Group2* (n=12): leg pain changes only  Age= 69 ± 8 yo  *Group3* (n=9): non-change  Age= 71 ± 9 yo | Velocity, peak trunk tilt, peak thoracic tilt, peak lumbar tilt, peak pelvic tilt | Changes in leg pain  Changes in low back pain | ***Changes in leg pain***  Moderate correlation between leg pain changes and peak trunk tilt pre-effort (r=-0.455; p=0.044).  There was no significant correlation between leg pain changes and velocity, peak trunk tilt post effort, trunk changes, peak thoracic tilt, peak lumbar tilt and peak pelvic tilt (r= [-0.206 - 0.286]).  ***Changes in low back pain***  Moderate correlation between low back pain changes and peak lumbar tilt pre (r=-0.508; p=0.022) and post (r=-0.503; p=0.024) effort and changes in pelvis variation (r=0.506; p=0.023).  There was no significant correlation between low back pain change and velocity, peak trunk tilt, peak thoracic tilt, changes in lumbar spine and peak pelvic tilt pre- and post-effort (r= [-0.274 - 0.343]). | Non-change group was excluded of the correlation. |
| **13** | **Minetama et al. 2020** | Associations between psychological factors and daily step count in patients with lumbar spinal stenosis | Japan | Secondary analysis of a RCT | 71 | **LSS:** LSS characterized by NC.  Diagnosis was based on clinical examination and MRI findings  **NC:** defined as leg pain, numbness, and heaviness brought on by walking but relieved by sitting. | Patients with LSS with NC (N=71)  M: F ratio = 35: 36  Age ± SD = 71.6 ± 5.6 | Age, sexe, BMI, number of stenoses, duration of symptoms, severity of stenosis, walking distance on the SPWT, ZCQ, NRS (leg and LBP), PCS, PASS-20 (total score, cognitive anxiety, escape/avoidance, fear), TSK-11 and HADS depression score | Daily step count | Strong correlation between daily step count and age (r = – 0.543 ; p<0.05), number of stenoses (r = 0.260 ; p<0.05), severity of stenosis at L3–L4 (r = 0.279 ; p<0.05), walking distance on the SPWT (r = 0.338 ; p<0.05), PASS-20 total score (r = – 0.327 ; p<0.05); cognitive anxiety (r = – 0.352 ; p<0.05); escape/avoidance (r = – 0.235 ; p<0.05); fear (r = – 0.284 ; p<0.05), and HADS depression score (r = – 0.245 ; p<0.05)  There was no significant correlation between daily step count and sexe, BMI, duration of symptoms, severity of stenosis (L1-L2, L2-L3,L4-L5,L4-S1), ZCQ, NRS (leg and LBP), PCS, and TSK-11 (p>0.05). |  |
| **14** | **Nagai et al. 2014** | Quantification of Changes in Gait Characteristics Associated With Intermittent Claudication in Patients With Lumbar Spinal Stenosis | Japan | Cross-sectional observational study | 11 | **LSS:** NA  Diagnosis was based on patient history, physical examination and MRI.  **NC:** Pain, numbness, weakness, or tingling in the lower extremities brought on by lumbar extension, standing, or walking | Patients with LSS with NC (N=11)  M: F ratio=  8: 3  Age= 72.8 ± 5.5 yo | RMS, autocorrelation coefficient, stride frequency, coefficient of variance | Maximum walking distance | Strong correlation between maximum walking distance and RMS at the cervical sensor (r=-0.64; p=0.03) and coefficient of variance (r=-0.66; p=0.03).  There was no significant correlation between maximum walking distance and RMS at the lumbar sensor, autocorrelation coefficient for both cervical and lumbar sensor position and stride frequency (r= [-0.55 - 0.53]). |  |
| **15** | **Özdermir et al 2015** | Quality of life and related factors in  degenerative lumbar spinal stenosis: A  controlled study | Turkey | Cross-sectional study (controlled) | 108 | **LSS:**  Lumbar spinal stenosis (LSS) is a common degenerative  disease that occurs narrowing of the spinal canal,  neural foramen and lateral recess due to changes in the  soft tissue or bone.  Diagnosis was based on clinical findings and MRI.  **NC:** NA | *Group 1* (n=54):  Patients with diagnosis of LSS by clinical findings and MRI  M: F ratio = 9: 45  Age= 68.06 ± 9.55 yo  *Group 2* (n=54): Healthy age and sex matched  M: F ratio =11: 43  Age = 67.15 ± 9.50 yo | ODI,  HADS,  Walking distance,  Comorbidities,  Duration of symptoms, Number of spinal segments with stenosis | **SF-36** (physical health, mental health and total) | ***Physical health***  Strong correlation between physical health and ODI (r= −0.713; p<0.001) and HADS-A (r= −0.583; p<0.001).  Moderate correlation between physical health and HADS-D (r= −0.479; p<0.001) and walking distance (r= 0.476; p<0.001).  ***Mental health***  Strong correlation between mental health and ODI (r= −0.638; p<0.001), HADS-A (r= −0.682; p<0.001) and HADS-D (r= −0.569; p<0.001).  Moderate correlation between mental health and walking distance (r= 0.319; p<0.05).  ***Total SF-36***  Strong correlation between total SF-36 score and ODI (r= −0.715; p<0.001), HADS-A (r= −0.627; p<0.001) and HADS-D (r= −0.522; p<0.001).  Moderate correlation between total SF-36 and walking distance (r= 0.458; p<0.05).  No correlation was found between the SF-36 scores and comorbidities, duration of symptoms and the number of spinal segments with stenosis. | *Age and sex matched pas respecté*  *Walking distance was not explained in the study.* |
| **16** | **Pryce et al. 2012** | Relationship Between Ambulatory Performance and Self-Rated Disability in Patients With Lumbar Spinal Stenosis | Canada | Cross-sectional study | 33 | **LSS:** NA  Diagnosis was based on symptoms and MRI.  **NC:** Activity-generated, neutrally mediated lower limb pain, paresthesia, or cramping. | Patients with LSS with NC (N=33)  M: F ratio = NA  Age= 68.5 ± 8.4 yo | SF-36, RMDQ, ODI, DASH, VAS (back and leg pain) | **Physical activity** (volume, intensity, duration)  **Ambulatory behavior** (bout length, maximum bout length)  **Sedentary behavior** (duration, bout length, maximum bout length) | ***Physical activity:***  There were significant moderate to strong correlations between PA volume and SF-36, disability and pain (r=[ -0.58 – 0.64].  There were significant moderate to strong correlations between PA intensity and SF-36 (total score, physical health, mental health and physical function) and disability (r= [-0.49 – 0.55]).  There was no significant correlation between PA intensity and SF-36 (bodily pain) and pain (back pain intensity, leg pain intensity, back pain function and leg pain function).  There were significant moderate to strong correlations between PA duration and SF-36, disability and pain (back pain intensity, back pain function and leg pain function (r= [-0.50 – 0.58]).  There was no significant correlation between PA duration and leg pain intensity (r= -0.26).  Regression models for physical activity showed that PA volume was predicted by SF-36 physical function and RMDQ (r^2^= 0.43; p= 0.001), intensity was predicted by SF-36 physical function (r^2^= 0.28; p= 0.001) and duration was predicted by SF-36 physical function (r^2^= 0.31; p= 0.01)  ***Ambulatory behavior (meaningful intensity)***  There were significant moderate to strong correlations between bout length and maximum bout length and SF-36, disability and back pain (r= [-0.51 – 0.73]. There were significant moderate correlations between maximum bout length and pain (back pain function (r= -0.39) and leg pain function (r= -0.43)).  Regression models for meaningful activity showed that bout length was predicted by SF-36 physical function (r^2^= 0.28; p=0.001) and that maximum bout length was also predicted by SF-36 physical function (r^2^ = 0.52; p< 0.001).  ***Ambulatory behavior (moderate intensity)***  There were significant moderate to strong correlation between bout length and SF-36 (total score, physical health, physical function and bodily pain), disability (RMDQ and ODI) and pain (leg pain intensity).  There was no significant correlation between bout length and SF-36 (mental health), disability (DASH) and pain (back pain intensity, back pain function and leg pain function) (r= [-0.30 – 0.32]).  There were significant moderate to strong correlation between maximum bout length and SF-36 (total score, physical health, mental health and physical function), disability (RMDQ, ODI and DASH) and leg pain intensity (r= [-0.55 – 0.61].  There was no significant correlation between maximum bout length and SF-36 (bodily pain) and pain (back pain intensity, back pain function and leg pain function) (r= [-0.34 – 0.33]).  Regression models for moderate activity showed that bout length was predicted by SF-36 physical function, leg pain intensity and back pain intensity (r^2^= 0.38; p< 0.01) and tht maximum bout length was predicted by SF-36 physical function and ODI (r^2^= 0.38; p< 0.001).  ***Sedentary behavior***  There was no correlation between dependant variables (duration, bout length and maximum bout length) and independent variables (SF-36, disability and pain) (r= [-0.34 – 0.32]).  Regression models for sedentary behavior showed that bout length was predicted by SF-36 physical function and back pain intensity (r^2^= 0.22; p= 0.01). There were no predictors for duration and maximum bout length. | *p<0.05 when r>0.35*  *p<0.01 when r>0.45* |
| **17** | **Quack et al. 2019** | Psychological factors outmatched morphological markers in predicting limitations in activities of daily living and participation in patients with lumbar stenosis | Germany | Cross-sectional | 67 | **LSS:** Degenerative lumbar spinal stenosis is related to narrowing of the lumbar spinal canal and is associated with degenerative changes of the joint complex, osteophyte formation and ligamentum flavum thickening.  Diagnosis was based on clinical evaluation and MRI.  **NC:** NA | Patients with degenerative LSS  (n=67)  M: F ratio = 34: 33  Age = 62.5 ± 11.7 yo | Age, gender, duration of disease, the three morphological markers (Schizas classification, number of stenotic segments and presence of a spondylolisthesis), depression and fear of movement | Pain interference (PI-G), performance of ADLs (RehaCAT subscales), participation (AAPI-Part) | ***PI-G:***  PI-G mental was strongly correlated with DESC (r= 0.55; p<0.01). PI-G mental was weakly correlated with number of stenotic segments (r= -0.28; p<0.05), TSK (SF: r= 0.28; p<0.05 and AA: r= 0.25; p<0.05).  There was no significant correlation between PI-G mental and age, gender, disease duration, spondylolisthesis and Schizas classification (r= [-0.16 – 0.22]).  PI-G functional was moderately correlated with DESC (r= 0.48; p<0.01) and TSK (AA: r= 0.30; p<0.05).  PI-G functional was weakly correlation with gender (r= 0.29; p<0.05 and number of stenotic segments (r=-0.25; p<0.05).  There was no significant correlation between PI-G functional and age, disease duration, spondylolisthesis, Schizas classification and TSK SF (r= [-0.19 - -0.01]).  PI-G physical was moderately correlated with DESC (r= 0.35; p<0.05) and was weakly correlated with TSK (SF: r= 0.26; p<0.05).  There was no significant correlation between PI-G physical and gender, age, disease duration, spondylolisthesis, number of stenotic segments, Schizas classification and TSK AA (r= [-0.11 – 0.24]).  ***RehaCAT:***  RehaCAT lower extremity was strongly correlated with TSK SF (r=0.52; p<0.01) and moderately correlated with Schizas classification (r= 0.32; p<0.05), DESC (r= 0.43; p<0.01) and TSK AA (r= 0.45; p<0.01).  There was no significant correlation between RehaCAT lower extremity and gender, age, disease duration, spondylolisthesis and number of stenotic segments (r =[ -0.17 – 0.20]).  RehaCAT upper extremity was strongly correlated with TSK SF (r=0.64; p<0.01) and moderately correlated with DESC (r= 0.40; p<0.01) and TSK AA (r=0.45; p<0.01).  There was no significant correlation between RehaCAT and age, gender, disease duration and morphological markers (r= [-0.03 – 0.23]).  RehaCAT activities of daily living was moderately correlated with DESC (r= 0.45; p<0.01) and TSK (SF: r= 0.43; p<0.01 and AA: r= 0.44; p<0.01). RehaCAT activities of daily living was weakly correlated with gender (r= 0.29; p<0.05) and disease duration (r= -0.27; p<0.05).  There was no significant correlation between RehaCAT activities of daily living and age, spondylolisthesis, number of stenotic segment and Schizas classification (r= [-0.18 – 0.10].  ***AAPI participation:***  AAPI participation subscale was strongly correlated with DESC (r= -0.57; p<0.01), moderately correlated with TSK SF (r= -0.39; p<0.01) and weakly correlated with number of stenotic segments (r= 0.26; p<0.05).  There was no significant correlation between AAPI participation and gender, age, disease duration, spondylolisthesis, Schizas classification and TSK AA (r= [-0.25 – 0.20]).  DESC alone predict PI-G mental (r^2^=0.32) and PI-G physical (r^2^= 0.12).  DESC combined with TSK AA predict PI-G functional (r^2^=0.28) and RehaCAT activities of daily living (r^2^= 0.38).  DESC combined with TSK SF predict AAPI participation (r^2^= 0.40).  DESC combined with TSK SF and Schizas classification predict RehaCAT lower extremity (r^2^= 0.39)  TSK SF alone predict RehaCAT upper extremity (r^2^=0.40). |  |
| **18** | **Schmidt et al. 2017a** | Association of Neuromuscular Attributes With Performance-Based Mobility Among Community-Dwelling Older Adults With Symptomatic Lumbar Spinal Stenosis | USA | Cross-sectional study | 300 | **LSS:** NA  Diagnosis was bas on the combination of imaging-detected LSS and self-reported NC.  **NC:** Pain, numbness or weakness in the lower extremities that worsens with spinal extension and improves with spinal flexion. | Patients with LSS  *Group1* (n=54): Patients with symptomatic LSS  Age = 76.9 ± 6.6 yo  *Group2* (n=246): Patients with no symptomatic LSS  Age = 77.1 ± 7.2 yo | Trunk extensor muscle endurance, leg strength, leg strength asymmetry, leg speed, maximum knee flexion ROM and asymmetry, maximum knee extension ROM and asymmetry, ankle ROM and kyphosis | **Performance-based measures of mobility** (SPPB total score, HGS and Chair Stand) | ***SPPB total score:***  Trunk extensor muscle endurance, leg strength asymmetry and leg speed were weakly associated with the SPPB score (r^2^= 0.24).  ***HGS:***    Trunk extensor muscle endurance, knee flexion ROM and knee extension asymmetry were weakly associated with HGS (r^2^= 0.35).  ***Chair stand test:***  Leg strength was weakly or poorly associated with Chair Stand test (r*^2^*= 0.19). |  |
| **19** | **Sigmundsson et al. 2011** | Correlation between disability and MRI findings in lumbar spinal stenosis | Sweden | Prospective study | 109 | **LSS:** dural sac area of 70 mm2, presence of NC, persistent leg and/or back pain and weakness and numbness in one or both legs.  **NC:** NA | Patients with a central spinal stenosis planned for a surgery  M:F ratio = 53: 56  Age = 71 ± 10 yo | Estimated walking distance, VAS (leg and back), ODI, SF-36 (bodily pain, PF, role physical, general health) and EQ-5D | Minimal dural sac area and multilevel stenosis | ***Minimal dural sac area:***  There was no significant correlation between minimal dural sac area estimated walking distance, VAS (leg and back), ODI, SF-36 (bodily pain, PF, role physical, general health) and EQ-5D) (r= [-0.11 – 0.13]).  ***Multilevel spinal stenosis:***  There was a weak correlation between VAS (leg) and multilevel spinal stenosis (r=-0.24; p=0.0).  There was no significant correlation between multilevel spinal stenosis and estimated walking distance, VAS (back), ODI, SF-36 (bodily pain, PF, role physical, general health) and EQ-5D) (r= [-0.14 – 0.10]). | Estimated walking distance was measured in 105 participants.  **Controlling for number of levels involved* |
| **20** | **Thornes et al. 2018a** | Degenerative lumbar spinal stenosis and physical functioning: an exploration of associations between self-reported measures and physical performance tests | Norway | Cross-sectional study | 103 | **LSS:** NA  Diagnosis was based on symptoms and radiological findings.  **NC:** NA | Patients with a symptomatic and radiological verified central spinal stenosis  M:F ratio = 48 : 55  Age= 71.3 ±7.3 yo | OLS in 30 seconds, tandem walking 2 m, functional leg strength (30-s SS), stair climbing (10 steps up and 10 steps down),  Duration of present lumbar pain, duration of present leg pain, HSCL25, age, gender and BMI | SYMP and FUNC scales from the SSSQ | ***SYMP scale:***  Moderate correlation between SYMP scale and 30-s SS (r= -0.32; p=0.01), stair climb (r= 0.40; p=0.01), OLS (r= -0.33; p=0.01), duration of present lumbar pain (r= 0.25; p=0.05), duration of present leg pain (r=0.31; p=0.01) and HSCL25 (r= 0.39; p=0.01).  There was no significant correlation between SYMP scale and tandem walk, age, gender and BMI (r= [-0.20 – 0.16]).  ***FUNC scale:***  Strong correlation between FUNC scale and stair climb (r= 0.58; p=0.01).    Moderate correlation between FUNC scale and 30/s SS (r= -0.44; p= 0.01), tandem walk (r= -0.29; p= 0.01), OLS (r= -0.28; p= 0.01), gender (r= -0.22; p= 0.05), duration of present lumbar pain (r= 0.26; p= 0.01) and HSCL25 (r= 0.50; p= 0.01).  There was no significant correlation between FUNC scale and age, BMI and duration of present leg pain (r= [0.04 – 0.18]). |  |
| **21** | **Thornes et al. 2018b** | Dynamic balance in patients with degenerative lumbar spinal stenosis; a cross-sectional study | Norway | Cross-sectional study | 62 | **LSS:** NA  Diagnosis was based on symptoms and radiological findings.  **NC:** NA | Patients with MRI-verified LSS.  M: F ratio = 33: 29  Age = 71.2 ± 7.1 yo | Mini-BESTest  **Control systems** (anticipatory adjustment, reactive response, sensory orientation and stability in gait) | FUNC scale and ODI | ***FUNC scale:***  Adjusted linear regression showed a significant association between FUNC scale and Mini-BESTest (b= -0.1; p= 0.042).  There were no significant association between FUNC scale and four control system.  Participants dynamic balance explain 16.7% of the variation in FUNC.  ***ODI:***  Adjusted linear regression showed a significant association between ODI and Stability in Gait (b= -5.3; p=0.001)  There was no significant association between ODI and Mini-BESTest and the others control systems. | Participants were sometimes divided in three or two groups (correlation, bivariate correlation). |
| **22** | **Tomkins-Lane, 2013** | Predictors of objectively measured walking capacity in people with degenerative lumbar spinal stenosis | Canada | Cross-sectional | 49 | **LSS:** Characterized by a narrow spinal canal and/or narrow nerve root foramina, resulting from degenerative changes in the spine.  Diagnosis was based on patient history, physical examination and MRI.  **NC:** Progressive onset of pain and neuromuscular deficit including numbness, tingling, and  weakness in the low back, buttocks, and legs.  These symptoms are initiated by standing or lumbar  extension and exacerbated by walking | Patients with LSS with NC or walking limitations (N=49)  M: F=  25: 24  Age= 65.8 ±10.0 yo | *Domains:* demographic (age and gender), pain location, duration of pain, pain severity, pain related disability, severity of neurological symptoms (numbness, weakness, tingling), location of neurological symptoms, balance problems and health-related quality of life  *Questionnaires used:* ODI, SSSQ, HUI, VAS | **Walking capacity** (SPWT distance) | Variable retained from the domain-specific regression models explaining walking capacity were: years of leg pain, pre-test leg pain severity (VAS), ODI, SSSQ (symptom severity subscale item #7) and HUI.  *Duration of pain:* Moderate correlation between walking capacity and years of back pain (r=0.29; p<0.05) and years of leg pain (r=0.42; p<0.01).  *Pain severity:* Moderate correlation between walking capacity and SSSQ symptom scale (r=-0.28; p<0.05) and pre-walk leg VAS (r=-0.32; p<0.05).  *Pain related function:* Strong correlation between walking capacity and ODI (r=0.52; p<0.01). Moderate correlation between walking capacity and HUI Q8 (r=-0.35; p<0.05) and HUI Q15 (r=-0.33; p<0.05).  Severity of neurologic symptoms: Moderate correlation between walking capacity and balance problems (r=-0.40; p<0.01).  Health related quality of life: Moderate correlation between walking capacity and HUI score (r=0.38; p<0.05). |  |
| **23** | **Tong et al. 2007** | Comparing Pain Severity and Functional Status of Older Adults without Spinal Symptoms, with Lumbar Spinal Stenosis, and with Axial Low Back Pain | USA | Cross-sectional | 48 | **LSS:** NA  **NC:** NA | *Group 1* (n=24): Patients with lumbar spinal stenosis  M: F 14: 10  Age = 68.6 ± 6.8 yo  *Group 2* (n=12): Patients with axial back pain  *Group 3* (n=12): asymptomatic | Pain (VAS), 15 min walk test and 7-day walking distance | PDI, QBPDS | Pain VAS was significantly related to QBPDS (β= 0.54; p=0.03) and to PDI (β= 0.76; p=0.002) in spinal stenosis patients.  There was no signification association between QBPDS or PDI with 15-min walk test and 7-day walking test. |  |
| **24** | **Zeifang et al. 2008** | Gait analysis does not correlate with clinical and MR imaging parameters in patients with symptomatic lumbar spinal stenosis | Germany | Cross-sectional | 63 | **LSS:**  Narrowing of the spinal canal.  Diagnosis was based on symptoms and MRI.  **NC:**  Limited walking distance (pain in lower extremities aggravated by walking and lumbar extension and alleviated with lumbar flexion) | Patients with symptomatic LSS (N=63)  M:F ratio = 26: 37  Age= 68 yo (median) | Demographic data (age, BMI), functional status, CES-D, cross-sectional area of the overall dural tube, number of segments with a cross-sectional area of less than 70 mm^2^ | Walking distance | A significant correlation was observed between the objectively measured walking distance and the BMI (tau b= -0.194; p=0.025), functional status (tau b=0.225; p=0.011) and the cross-sectional area of the dural tube at L1/2 (tau b=-0.118; p=0.032).  There was no significant correlation between walking distance and depression status (CES-D). |  |

LSS= Lumbar Spinal Stenosis, NC= Neurogenic Claudication, ODI= Oswestry Disability Index, NRS= Numeric Rating Scale, SF-12= Medical Outcomes Short-Form 12, SPWT= Self-Paced Walking Test, QBPDS= Quebec Back Pain Disability Scale, SSSQ= Swiss Spinal Stenosis Questionnaire, SF-36= 36-item Short Form Health Survey, JOABPEQ= Japanese Orthopedic Association Back Pain Evaluation Questionnaire, ZCQ= Zurich Claudication Questionnaire, TUGT= Timed Up-and-Go Test, GDI= Gait Deviation Index, VAS= Visual Analog Scale, MRI= Magnetic Resonance Imaging, BDI= Beck Depression Inventory, EMG= Electromyography, RMS= Root Mean Square, HADS= Hospital Anxiety and Depression Scale, RMDQ = Roland-Morris Disability Questionnaire, DASH= Disability of the Arm, Shoulder and Hand questionnaire, ROM= Range of Motion, HGS= Habitual Gait Speed, DESC= Rasch-based Depression Screener, TSK= Tampa Scale of Kinesiophobia, AA= Activity Avoidance, SF= Somatic Focus, ADL=Activity of Daily Living, RehaCAT = Rasch-based RehaCAT-system, PASS-20 = Pain Anxiety Symptoms Scale, PCS = Pain Catastrophizing Scale, PI-G= Pain Interference Scale-German, SPPB= Short Physical Performance Battery, SYMP scale = Symptom severity scale of the SSSQ, FUNC scale = Physical function scale of the SSQ, OLS= One Leg Stance test, 30-s SS= 30 seconds Sit to Stand, HSCL25= Hopkins symptom check list, HUI= Health Utilities Index Mark 3, PDI= Pain Disability Index, CES-D= Center of Epidemiological Studies Depression Scale,
