# Supplementary material for: Physical and Psychological Factors Associated With Walking Capacity in Patients With Lumbar Spinal Stenosis With Neurogenic Claudication: A Systematic Scoping Review
Source: Front Neurol. 2021 Sep 9;12:720662. doi: 10.3389/fneur.2021.720662 (PMC8459720; doi:10.3389/fneur.2021.720662)
Supplement: Supplementary file 3 [file Data_Sheet_3.pdf]

Quality assessment (Appraisal tool for Cross-Sectional Studies (AXIS))

Study/Criteria	1.	2.	3.	4	5.	6.	7.	8.	9.	10.	11.	12.	13.	14.*	15.	16.	17.	18.	19.**	20.	Total score
**Conrad et al. (2013)**	1	1	0	0	1	1	0	1	1	1	1	1	0	NA	1	1	1	1	1	1	15
**Conway et al. (2010)**	1	1	0	1	1	1	0	1	1	1	1	1	0	NA	1	1	1	1	1	1	16
**Drury et al. (2009)**	1	1	1	1	1	1	0	1	1	1	1	0	0	NA	1	1	1	1	1	1	16
**Fujita et al. (2019)**	1	1	0	1	1	1	0	1	1	1	1	1	0	NA	1	1	1	1	1	1	16
**Gaberlotti et al. (2014)**	1	1	0	1	1	1	0	1	1	1	1	1	0	NA	1	1	1	1	1	1	16
**Grelat et al. (2018)**	1	1	0	1	1	1	0	1	1	1	1	1	0	NA	1	1	1	1	1	1	16
**Igawa et al. (2018)**	1	1	0	1	1	1	0	1	1	1	1	1	0	NA	1	1	1	1	1	1	16
**Inoue et al. (2020)**	1	1	0	1	1	1	0	1	0	1	0	1	0	NA	1	-	1	1	1	1	13
**Ishimoto et al. (2012)**	1	1	0	0	1	1	0	1	1	0	0	1	0	NA	1	1	1	1	1	1	13
**Kim et al. (2011)**	1	1	0	1	1	1	0	1	1	1	1	1	0	NA	1	1	1	1	1	0	15
**Kuittinen et al. (2014)**	1	1	0	1	1	0	0	1	1	1	1	0	0	NA	1	0	1	1	1	1	13
**Kuwahara et al. (2018)**	1	1	0	0	1	1	0	1	1	1	1	1	0	NA	1	1	1	1	1	1	15
**Minetama et al. (2020)**	1	1	0	0	1	1	0	1	1	1	1	1	0	NA	1	1	1	1	1	1	15
**Nagai et al (2013)**	1	1	0	1	0	0	0	1	-	1	0	1	0	NA	1	1	1	1	1	1	12
**Özdemir et al. (2015)**	1	1	0	0	1	0	0	1	1	1	0	0	0	NA	-	1	1	1	1	1	11
**Pryce et al. (2012)**	1	1	1	0	1	1	0	1	1	1	1	-	0	NA	1	1	1	1	1	1	15
**Quack et al. (2019)**	1	1	0	1	1	1	0	1	1	0	1	1	0	NA	0	1	1	1	1	1	14
**Schmidt et al. (2017)**	1	1	0	1	1	1	0	1	1	1	0	1	0	NA	0	1	1	1	1	0	13
**Sigmundsson et al. (2011)**	1	1	0	0	1	-	0	1	0	1	0	0	0	N/A	0	1	1	0	1	1	9
**Thornes et al. a (2018)**	1	1	0	1	1	1	0	1	1	1	1	1	0	NA	1	1	1	1	1	1	16
**Thornes et al. b (2018)**	1	1	0	1	1	1	0	1	1	1	1	1	0	NA	1	1	1	1	1	1	16
**Tomkins et al. (2013)**	1	1	0	1	1	0	0	1	1	1	1	1	-	NA	1	0	1	1	1	1	14
**Tong et al. (2006)**	1	1	0	0	-	0	0	0	1	0	0	1	0	NA	1	1	1	1	1	1	10
**Zeifang et al. (2008)**	1	1	0	1	1	0	0	1	1	1	1	1	0	NA	1	1	1	1	1	0	14
**Total (nb of "yeses" per questi**	24	24	2	16	22	17	0	23	21	21	17	19	0	N/A	20	21	24	23	24	21	

*Question 14 was removed of the total score because the question is not applicable

**This question was given one point when it was specified that there was no conflict of interest

Legend: 1 = yes ; 0 = no; – = do not know; NA = not applicable

Blue (Quality of reporting) = Questions 1, 4, 10, 11, 12, 16, and 18

Orange (Study design quality) = Questions 2, 3, 5, 8, 17, 19, and 20

Green (Risk of bias) = Questions 6, 7, 9, 13, 14, and 15

Questions/Criteria

Introduction

1. Were the aims/objectives of the study clear?

Methods

2. Was the study design appropriate for the stated aim(s)?
3. Was the sample size justified?
4. Was the target/reference population clearly defined? (Is it clear who the research was about?)
5. Was the sample frame taken from an appropriate population base so that it closely represented the target/reference population under investigation?
6. Was the selection process likely to select subjects/participants that were representative of the target/reference population under investigation?
7. Were measures undertaken to address and categorise non-responders?
8. Were the risk factor and outcome variables measured appropriate to the aims of the study?
9. Were the risk factor and outcome variables measured correctly using instruments/measurements that had been trialled, piloted or published previously?
10. Is it clear what was used to determined statistical significance and/or precision estimates? (e.g. p-values, confidence intervals)
11. Were the methods (including statistical methods) sufficiently described to enable them to be repeated?

Results

12. Were the basic data adequately described?
13. Does the response rate raise concerns about non-response bias?
14. If appropriate, was information about non-responders described?
15. Were the results internally consistent?
16. Were the results presented for all the analyses described in the methods?

Disusion

17. Were the authors' discussions and conclusions justified by the results?
18. Were the limitations of the study discussed?

Other

19. Were there any funding sources or conflicts of interest that may affect the authors’ interpretation of the results?
20. Was ethical approval or consent of participants attained?
