# Supplementary material for: Physical and Psychological Factors Associated With Walking Capacity in Patients With Lumbar Spinal Stenosis With Neurogenic Claudication: A Systematic Scoping Review
Source: Front Neurol. 2021 Sep 9;12:720662. doi: 10.3389/fneur.2021.720662 (PMC8459720; doi:10.3389/fneur.2021.720662)
Supplement: Supplementary file 4 [file Data_Sheet_4.docx]

**Supplement 4: Non-significant correlations reported in the 22 included studies.**

*Walking Capacity*

PROMS

**Pain**

Conway et al. 2011 reported no significant correlation between leg pain severity and walking distance to first symptoms or total walking distance and Tong et al. 2007 reported no significant correlation when walking capacity measured with the 15-min walking test of the 7-day walking test [1, 2]. Ishimoto reported no significant association between back or leg pain and walking time at usual pace [3].

**Disability**

Two studies reported that there is no correlation between the total QBPDS score and walking distance [2, 4].

In the study of Conway and colleagues, the subscale “stand” was not significantly correlated to the total walking distance [1].

One study did not report a significant correlation between SSSQ physical function and symptoms subscales and distance to first symptoms measured with the Self-Paced Walking Test (SPWT) [1]. In addition, the symptom subscale of the SSSQ did not seem to be correlated with walking capacity when measured using the tandem walk test [5].

Conway et al. 2011 showed no significant correlation between walking distance to first symptoms and disability using the ODI [1].

**Quality of life**

One study using the SF-12 showed that the total walking perimeter was not correlated to this shorter version of the questionnaire [4]. In addition, walking distance before the first symptoms was not correlated with the PF subscale [1]. One study showed a non-significant correlation between mental health subscale and walking distance [6].

Objectives measures

**MRI findings**

Results showed that there was no significant correlation between measured or estimated walking distance and MRI findings such as severity of the entrance stenosis, mid zone, entrance zone width, mid zone area, dural sac area, or the number of levels with stenosis [7, 8].

**Other Objectives Measures**

One study showed that there was no significant correlation between activity (count per day) and walking capacity [1].

Another study did not show significant correlations with lumbar postural sway, autocorrelation coefficient for both cervical and lumbar sensors position and stride frequency [9].

Another study showed that there were no correlations between estimated walking distance and the minimal dural sac area, multilevel stenosis and low-grade spondylolisthesis [8] .

*Gait pattern characteristics*

PROMS

**Pain severity**

When assessing pain with the VAS, no correlation was found between pain and gait patterns characteristics [10, 11]. However, pain location in the study of Gaberlotti and colleagues was not defined. It was not possible to identify if pain perception was about back, leg or global pain [10]. Also, there was no significant correlation between pain perception pre-walking and kinematic parameters such as stride length, single stance, double stance, swing and trunk tilt or between leg pain changes and peak trunk tilt post effort, trunk changes, peak thoracic tilt, peak lumbar tilt and peak pelvic tilt [10, 11]. Moreover, there was no significant correlation between low back pain change and peak trunk tilt, peak thoracic tilt, variation of lumbar spine tilt and peak pelvic tilt pre- and post-effort [11].

Pain severity assessed using NRS was not significantly correlated to step length, lumbar ROM and lumbar proprioception. Cadence was not associated with pain regardless of the tool used [12].

Objectives Measures

There were no significant correlations between maximum trunk flexion angle and velocity or gait cycle time, maximum ankle dorsi flexion moment, maximum ankle plantar flexion angle, maximum ankle plantar flexion angle, maximum ankle power, and minimum ankle power [13].

*Functional task*

PROMS

**Pain**

There were no significant correlations between back or leg pain intensity and physical activity intensity [14].

There were no significant association between the function scale and the four control systems in balance control (anticipatory postural adjustments, reactive responses, sensory orientation and stability in gait) measured with the 14 tasks from the Mini-BESTest [15].

One study reported no significant association between pain (back, leg and numbness) and daily step count (MINE).

**Disability**

Most of the QBPDS subscales (total, stand, walk, reach and groceries) were not significantly correlated with activity count per day and time of continuous activity [1].

One study reported no association between the ZCQ also known as the SSSQ and daily step count [16].

Two studies did not show significant correlations between the ODI and functional task measured using activity count per day, maximum of continuous activity and the four functional mobility tests (Alternative-Step Test, 6-Meter-Walk Test, Sit-to-Stand test and Timed Up and Go test) [1, 17].

**Quality of life**

Two studies showed that the SF-36 was not correlated with activity count per day and maximum time of continuous activity as well as bout length (only mental health subscale) and maximum bout length (only bodily pain subscale) at moderate intensity [1, 14].

**Depression and fear avoidance**

Regarding results of Quack and colleagues, there were no significant correlations between participation and the activity avoidance subscale of the TSK [18].

Minetama and colleagues reported no significant correlation between daily step count and anxiety, and pain catastrophizing [16].

**References:**

1. Conway, J., C.C. Tomkins, and A.J. Haig, *Walking assessment in people with lumbar spinal stenosis: capacity, performance, and self-report measures.* The Spine Journal, 2011. **11**(9): p. 816-823.

2. Tong, H.C., et al., *Comparing pain severity and functional status of older adults without spinal symptoms, with lumbar spinal stenosis, and with axial low back pain.* Gerontology, 2007. **53**(2): p. 111-115.

3. Ishimoto, Y., et al., *Prevalence of symptomatic lumbar spinal stenosis and its association with physical performance in a population-based cohort in Japan: the Wakayama Spine Study.* Osteoarthritis Cartilage, 2012. **20**(10): p. 1103-8.

4. Grelat, M., et al., *Walking Speed as an Alternative Measure of Functional Status in Patients with Lumbar Spinal Stenosis.* World neurosurgery, 2019. **122**: p. e591-e597.

5. Thornes, E., H.S. Robinson, and N.K. Vøllestad, *Degenerative lumbar spinal stenosis and physical functioning: an exploration of associations between self-reported measures and physical performance tests.* Disability and rehabilitation, 2018. **40**(2): p. 232-237.

6. Drury, T., et al., *Degenerative spondylolisthesis in patients with neurogenic claudication effects functional performance and self-reported quality of life.* Spine, 2009. **34**(25): p. 2812-2817.

7. Kuittinen, P., et al., *Correlation of lateral stenosis in MRI with symptoms, walking capacity and EMG findings in patients with surgically confirmed lateral lumbar spinal canal stenosis.* BMC musculoskeletal disorders, 2014. **15**(1): p. 247.

8. Sigmundsson, F.G., et al., *Correlation between disability and MRI findings in lumbar spinal stenosis: a prospective study of 109 patients operated on by decompression.* Acta orthopaedica, 2011. **82**(2): p. 204-210.

9. Nagai, K., et al., *Quantification of changes in gait characteristics associated with intermittent claudication in patients with lumbar spinal stenosis.* Clinical Spine Surgery, 2014. **27**(4): p. E136-E142.

10. Garbelotti Jr, S.A., et al., *An investigation of the value of tridimensional kinematic analysis in functional diagnosis of lumbar spinal stenosis.* Gait & posture, 2014. **40**(1): p. 150-153.

11. Kuwahara, W., et al., *Correlation between spinal and pelvic movements during gait and aggravation of low back pain by gait loading in lumbar spinal stenosis patients.* Journal of Orthopaedic Science, 2019. **24**(2): p. 207-213.

12. Conrad, B.P., et al., *Associations of self-report measures with gait, range of motion and proprioception in patients with lumbar spinal stenosis.* Gait & posture, 2013. **38**(4): p. 987-992.

13. Igawa, T., et al., *Kinetic and kinematic variables affecting trunk flexion during level walking in patients with lumbar spinal stenosis.* Plos one, 2018. **13**(5): p. e0197228.

14. Pryce, R., et al., *Relationship between ambulatory performance and self-rated disability in patients with lumbar spinal stenosis.* Spine, 2012. **37**(15): p. 1316-1323.

15. Thornes, E., H.S. Robinson, and N.K. Vøllestad, *Dynamic balance in patients with degenerative lumbar spinal stenosis; a cross-sectional study.* BMC musculoskeletal disorders, 2018. **19**(1): p. 192.

16. Minetama, M., et al., *Associations between psychological factors and daily step count in patients with lumbar spinal stenosis.* Physiother Theory Pract, 2020: p. 1-9.

17. Kim, H.-J., et al., *The risk assessment of a fall in patients with lumbar spinal stenosis.* Spine, 2011. **36**(9): p. E588-E592.

18. Quack, V., et al., *Psychological factors outmatched morphological markers in predicting limitations in activities of daily living and participation in patients with lumbar stenosis.* BMC musculoskeletal disorders, 2019. **20**(1): p. 1-9.
