# Supplementary material for: Physical and Psychological Factors Associated With Walking Capacity in Patients With Lumbar Spinal Stenosis With Neurogenic Claudication: A Systematic Scoping Review
Source: Front Neurol. 2021 Sep 9;12:720662. doi: 10.3389/fneur.2021.720662 (PMC8459720; doi:10.3389/fneur.2021.720662)
Supplement: Supplementary file 5 [file Data_Sheet_5.docx]

**Tests used to assess walking capacity**

The assessment of walking capacity (walking time or distance) was conducted using highly heterogeneous tests across the included studies. For this reason, we decided to look at the validity and reliability of these different walking tests in the LSS population.

Table 1: Validity and reliability of walking tests

| **Walking Test** | **SPWT**  **(30 min)** | **SPWT**  **(15 min)** | **SWT** | **TWT**  **(0-1000m)** | **TWT**  **(20 min)** | **6-MWT** | **SMWT** | **50-meter walkway** | **Tandem Walk (2m)** |
| --- | --- | --- | --- | --- | --- | --- | --- | --- | --- |
| **Validity** | **✔** | **?** | **X** | **X** | **X** | **?** | **✔** | **X** | **X** |
| **Reliability** | **✔** | **?** | **✔** | **X** | **✔** | **✔** | **✔** | **X** | **X** |

SPWT= Self-Paced Walking Test, SWT= Shuttle Walking Test, TWT= Treadmill Walking Test, 6-MWT= 6-Minute Walking Test, SMWT= Six Meters Walk Test

Most of the walking tests used were not validated in individuals with symptomatic LSS. However, several were at least validated in elderly.

**Validity and/or reliability**

**SPWT (30 min):** (1, 2)

**SWT:** (3)

**TWT:** (20 min): (4)

**6-MWT:** (5)

**SMWT:** (6)

**References :**

1. Rainville J, Childs LA, Peña EB, Suri P, Limke JC, Jouve C, et al. Quantification of walking ability in subjects with neurogenic claudication from lumbar spinal stenosis--a comparative study. Spine J. 2012;12(2):101-9.

2. Tomkins CC, Battié MC, Rogers T, Jiang H, Petersen S. A criterion measure of walking capacity in lumbar spinal stenosis and its comparison with a treadmill protocol. Spine. 2009;34(22):2444-9.

3. Pratt RK, Fairbank JC, Virr A. The reliability of the Shuttle Walking Test, the Swiss Spinal Stenosis Questionnaire, the Oxford Spinal Stenosis Score, and the Oswestry Disability Index in the assessment of patients with lumbar spinal stenosis. Spine. 2002;27(1):84-91.

4. Deen HG, Zimmerman RS, Lyons MK, Mcphee MC, Verheijde JL, Lemens SM, editors. Test-retest reproducibility of the exercise treadmill examination in lumbar spinal stenosis. Mayo Clinic Proceedings; 2000: Elsevier.

5. Harada ND, Chiu V, Stewart AL. Mobility-related function in older adults: assessment with a 6-minute walk test. Archives of physical medicine and rehabilitation. 1999;80(7):837-41.

6. Kim H-J, Chun H-J, Han C-D, Moon S-H, Kang K-T, Kim H-S, et al. The risk assessment of a fall in patients with lumbar spinal stenosis. Spine. 2011;36(9):E588-E92.
