# Supplementary material for: Physical and Psychological Factors Associated With Walking Capacity in Patients With Lumbar Spinal Stenosis With Neurogenic Claudication: A Systematic Scoping Review
Source: Front Neurol. 2021 Sep 9;12:720662. doi: 10.3389/fneur.2021.720662 (PMC8459720; doi:10.3389/fneur.2021.720662)
Supplement: Supplementary file 6 [file Table_1.pdf]

Supplementary Table 1: Data extraction regarding associations between physical and psychological factors, and the three outcome categories (walking capacity, functional tasks, and gait pattern characteristics).

| First author, year | N | Participants included in associations | Associations with walking capacity | Associations with gait pattern characteristics | Associations with functional tasks |
|---|---|---|---|---|---|
| **Conrad et al. 2013** | 25 | Patients with symptomatic LSS<br>M: F ratio = 11: 14<br>Age ± SD= 62 ± 14 yo | Physical factors<br>NA<br><br>Psychological factors<br>NA | Physical factors<br><br>***Pain***<br>**Velocity – NRS ($r^2$=0.189; p=0.049)**<br>**Base of support – NRS ($r^2$=0.219; p=0.032)**<br>Step length, cadence, ROM and proprioception – NRS ($r^2$= [0.000 - 0.177] ; p>0.05)<br><br>***Disability***<br>**Step length $r^2$ – ODI =0.58; p<0.001)**<br>**Gait velocity – ODI ($r^2$=0.506; p=0.001)**<br>**Base of support – ODI ($r^2$=0.363; p=0.008)**<br>**Left lateral bending proprioception – ODI ($r^2$=0.213; p=0.036).**<br>Cadence – ODI ($r^2$=0.182 ; p=0.057)<br>ROM – ODI ($r^2$= [0.024; 0.163] ; p>0.05)<br>Proprioception (extension, flexion and right lateral bending) – ODI ($r^2$= [-0.053; -0.039] ; p> 0.05).<br><br>***QoL***<br>SF-12 – biomechanical measurements (velocity, cadence, step length, base of support, proprioception, ROM) ($r^2$ = [0.001 - 0.183] ; p>0.05).<br><br>Psychological factors<br>NA | Physical factors<br>NA<br><br>Psychological factors<br>NA |
| **Conway et al. 2011** | 12 | Patients with LSS with NC<br>M: F ratio = 9: 3<br>Age ± SD = 66.3 ± 9.8 yo | Physical factors<br><br>***Pain***<br>SPWT distance – leg pain (r=0.492 ; p>0.05)<br>SPWT time to first symptoms – leg pain (r=0.216 ; p>0.05)<br><br>***Disability***<br>**SPWT distance – QBPDS (total: r=-0.638; p<0.05, walk: r=-0.755; p<0.01, reach: r=-0.650; p<0.05, run: r=-0.664;** | Physical factors<br>NA<br><br>Psychological factors<br>NA | Physical factors<br><br>***Pain***<br>**Activity count per day – leg pain (r=0.623; p<0.05)**<br>**Maximum time of continuous activity per day – leg pain (r=0.754; p<0.01)**<br><br>***Disability***<br>**Activity count per day – QBPDS run (r=-0.884; p<0.01)**<br>Activity count per day – QBPDS (total score, stand, walk, reach and groceries r=[-0.291 to -0.065] ; p>0.05) |

| | | | | | |
|---|---|---|---|---|---|
| | | | **p<0.05, groceries: r=-0.727; p<0.05**, stand (r= -0.551 ; p>0.05)<br>**SPWT time to first symptoms – QBPDS stand (r=-0.681; p<0.05,** total score r=-0.549 ; p>0.05, walk: r=-0.439 ; p>0.05, reach: r=-0.436 ; p>0.05, run: r=-0.151 ; p>0.05, groceries: r=-0.628 ; p>0.05)<br><br>**SPWT distance – SSSQ (PF: r=-0.610; p<0.05, PF walk: r=-0.715; p<0.05, SS weakness: r=-0.742; p<0.01, SS balance: r=-0.673; p<0.05)**<br>**SPWT time to first symptoms – SSSQ (PF walk: r=-0.622; p<0.05, SS balance: r=-0.646; p<0.05,** weakness (r=-0.463 ; p>0.05)<br><br>**SPWT distance – ODI (r=-0.595; p<0.05)**<br>SPWT time to first symptoms – ODI (r=-0.310 ; p>0.05)<br><br>***QoL***<br>**SPWT distance – SF-36 physical functioning (r=0.825; p<0.01)**<br>SPWT time to first symptoms – SF-36 physical functioning (r=0.325 ; p>0.05)<br><br>***Other self-reported outcomes***<br>Strong relationship between SPWT distance and estimated walking distance (r=0.886; p<0.01).<br>Strong relationship between SPWT time to first symptoms and estimated walking (r=0.659; p<0.05)<br><br>***Other objectives measures***<br>**SPWT distance – maximum time of continuous activity per day (r=0.629; p<0.05)**<br>**Activity count per day – maximum time of continuous activity per day (r=0.879; p<0.01)**<br>Activity count per day – self-reported walking capacity (r=0.304 ; p>0.05)<br>Activity count per day – SPWT distance (r=0.527 ; p>0.05)<br>Activity count per day – SPWT time to first symptoms (r=-0.075 ; p>0.05)<br><br>Maximum time of continuous activity per day – SPWT time to first symptoms (r=0.344 ; p>0.05) | | **Maximum time of continuous activity per day – QBPDS run (r=-0.825; p<0.01)**<br>Maximum time of continuous activity per day – QBPDS (total score, stand, walk, reach and groceries r=[-0.282 - -0.162] ; p>0.05)<br><br>Activity count per day per day– ODI (r=-0.148 ; p>0.05)<br>Maximum time of continuous activity – ODI (r=-0.070 ; p>0.05)<br><br>Activity count per day – SSSQ (PF walk, SS weakness and SS balance r=[-0.249 – 0.127] ; p>0.05)<br>Maximum time of continuous activity per day– SSSQ (PF walk, SS weakness and SS balance r=[-0.375 - -0.072] ; p>0.05)<br><br>***QoL***<br>Activity count per day – SF-36 (r= 0.521 ; p>0.05)<br>Maximum time of continuous activity – SF-36 (r= 0.500 ; p>0.05)<br><br>***Other objectives measures***<br>**Activity count per day – maximum time of continuous activity (r= 0.879 ; p<0.01)**<br><br>**<u>Psychological factors</u>**<br>NA |

| | | | | | |
|---|---|---|---|---|---|
| | | | Maximum time of continuous activity per day – self-reported walking capacity (r=0.265 ; p>0.05)<br><br><br>**<u>Psychological factors</u>**<br>NA | | |
| **Drury et al. 2009** | 77 | Patients with NC secondary to LSS<br>M: F ratio = 39: 38<br><br>LSS without spondylolisthesis (n=32)<br>Age ± SD = 67.1 ± 9.8 yo<br><br>LSS with fixed spondylolisthesis (n=22)<br>Age ± SD = 70.4 ± 10.9 yo<br><br>LSS with mobile spondylolisthesis (n=23)<br>Age ± SD = 68.6 ± 10.8 yo | **<u>Physical factors</u>**<br><br>***Disability***<br>**Walking distance – SSSQ (total score: r=-0.50; p<0.001, physical function (r= -0.52; p<0.001), pain: r= -0.27; p=0.019, sensory: r=-0.36; p=0.002, neuroischemic: r= -0.30; p=0.009)**<br><br>***QoL***<br>**Walking distance – SF-36 physical functioning (r= 0.60; p<0.001)**<br>**Walking distance – SF-36 (role physical: r= 0.35; p=0.003, bodily pain: r=0.39; p=0.001, general health index: r=0.26; p=0.033 and social functioning: r=0.31; p=0.009)**<br>**Walking distance – SF-36 (vitality (r= 0.24; p=0.041),** role emotional and mental health index subscales (r= [0.06 - 0.075] ; p>0.05)<br><br>**<u>Psychological factors</u>**<br>NA | **<u>Physical factors</u>**<br>NA<br><br>**<u>Psychological factors</u>**<br>NA | **<u>Physical factors</u>**<br>NA<br><br>**<u>Psychological factors</u>**<br>NA |

| | | | | | |
|---|---|---|---|---|---|
| **Fujita et al. 2019** | 357 | Patients who planned to undergo primary surgery for LSS with leg pain/NC<br><br>M:F ratio= 201: 156<br>Age± SD = 73.3 ±5.47 yo | **Physical factors**<br>NA<br><br>**Psychological factors**<br>NA | **Physical factors**<br><br>***Disability***<br>**Short stride – JOABPEQ lumbar function <60 (OR=2.9 ; p<0.01)**<br>**Short stride – score of walking ability in JOABPEQ <30 (OR=1.6 ; p=0.04)**<br>**Short stride – score of social life in JOABPEQ <45 (OR=1.6 ; p=0.04)**<br>**Short stride – score of psychological disorder in JOABPEQ <45 (OR=1.9 ; p<0.01)**<br><br>**Short stride – symptom severity in ZCQ ≥4 (OR=1.8 ; p=0.03)**<br>**Short stride – physical function in ZCQ ≥2.5 (OR=2.0 ; p<0.01)**<br><br>**Short stride – motor deficit (OR=3.1 ; p<0.01)**<br>**Short stride – sagittal vertical axis of ≥50 mm (OR=2.4)**<br><br>**Psychological factors**<br>NA | **Physical factors**<br>NA<br><br>**Psychological factors**<br>NA |
| **Gaberlotti et al. 2014** | 14 | Patients with LSS<br><br>M:F ratio= 10: 4<br>Age ± SD = 74.5 ±9.8 yo | **Physical factors**<br>NA<br><br>**Psychological factors**<br>NA | **Physical factors**<br><br>***Pain***<br>**Post-effort pain perception – GDI (left side: r=-0.64; p<0.05 and right side: r=-0.53; p<0.05)**<br>Pain perception pre-effort – kinematic parameters (speed, cadence, stride length, single stance, double stance, swing and trunk tilt) (r= [-0.45; 0.46] ; p>0.05)<br>Pain perception post-effort – other kinematic parameters(speed, cadence, stride length, single or double stance, swing, trunk tilt, GDI) (r= [-0.42 - 0.19] ; p>0.05)<br><br>**Psychological factors**<br>NA | **Physical factors**<br>NA<br><br>**Psychological factors**<br>NA |
| **Grelat et al. 2019** | 38 | Patients with LSS referred to the neurosurgery department, with the ability to walk 10m or more without help<br><br>M: F ratio= 23: 15 | **Physical factors**<br><br>***Disability***<br>**Measured walking perimeter – ODI (r= -0.44; p= 0.005)**<br>Measured walking perimeter – QBPDS (r= -0.31; p= 0.06) | **Physical factors**<br><br>***Disability***<br>**Free walking speed – ODI (r= -0.71; p=0.001)**<br>**Free walking speed – QBPDS (r= -0.5; p= 0.0008)** | **Physical factors**<br>NA<br><br>**Psychological factors**<br>NA |

| | | | | | |
|---|---|---|---|---|---|
| | | Age ± SD = 69.3 ±7.9 yo | ***QoL***<br>SF-12 – measured walking perimeter (p>0.05)<br><br>**Psychological factors**<br>NA | **Psychological factors**<br>NA | |
| **Igawa et al. 2018** | 111 | Patients with acquired degenerative LSS with leg pain<br><br>M: F ratio= 65: 51<br>Age ± SD = 70.9 ±6.2 yo | **Physical factors**<br>NA<br><br>**Psychological factors**<br>NA | **Physical factors**<br><br>***Objective measures***<br>**Anterior trunk flexion angle – step length (r=0.17; p=0.01)**<br>**Anterior trunk flexion angle – maximum ankle plantar flexion moment (r=0.20; p<0.01)**<br><br>Maximum trunk flexion angle – velocity or gait cycle time, maximum ankle dorsi flexion moment, maximum ankle plantar flexion angle, maximum ankle plantar flexion angle, maximum ankle power, and minimum ankle power (r=[-0.11 - 0.12] ; p>0.05)<br><br>**Psychological factors**<br>NA | **Physical factors**<br>NA<br><br>**Psychological factors**<br>NA |
| **Inoue et al. 2020** | 183 | Patients with LSS NC or radicular leg pain<br><br>M:F ratio = 128: 55<br>Age = 70.5 yo (range 36-88 yo) | **Physical factors**<br><br>***Objective measures***<br>**Handgrip strength – walking time (r= -0.269 ; p<0.001)**<br>**Handgrip strength – intermittent claudication (measure with the 300 m walking distance test) (r=0.201 ; p=0.008)**<br><br>**Psychological factors**<br>NA | **Physical factors**<br>NA<br><br>**Psychological factors**<br>NA | **Physical factors**<br><br>***Objective measures***<br>**Handgrip strength – walking (step) (r= -0.352 ; p<0.001)**<br><br>**Psychological factors**<br>NA |
| **Ishimoto et al. 2012** | 1009 | Total study<br>Age ± SD = 66.3 ±13.6 yo<br><br>*Group 1:* symptomatic LSS (N= 94)<br>M:F =34:60 | **Physical factors**<br><br>***Pain***<br>**Back and/or leg pain – 6-m walking time at maximal pace (OR = 1.17 ; p<0.05)**<br>Back and/or leg pain – 6-m walking time at usual pace (OR=1.04 ; p>0.05)<br><br>**Psychological factors**<br>NA | **Physical factors**<br>NA<br><br>**Psychological factors**<br>NA | **Physical factors**<br><br>***Pain***<br>Back and/or leg pain – chair standing time (OR= 1.03 ; p>0.05)<br>Back and/or leg pain – one-leg standing time (OR =1.00 ; p>0.05)<br><br>**Psychological factors**<br>NA |
| **Kim et al. 2011** | 80 | Patients with LSS with NC<br>(n=40) | **Physical factors**<br>NA | **Physical factors**<br>NA | **Physical factors**<br><br>***Disability*** |

| | | | | | |
|---|---|---|---|---|---|
| | | M: F ratio= 11: 29<br>Age ± SD = 62.82 ±7.29 yo | **<u>Psychological factors</u>**<br>NA | **<u>Psychological factors</u>**<br>NA | ODI – four functional mobility tests (alternative-step test, six-meter walk, sit-to-stand, timed-up and go) (p>0.05)<br><br>**<u>Psychological factors</u>**<br>NA |
| **Kuittinen et al. 2014** | 14 | Patients with LSS with leg pain and/or NC<br><br>M:F ratio= 6: 8<br>Mean age = 58 yo (range 48-76) | **<u>Physical factors</u>**<br><br>***Objective measures***<br>MRI findings – walking distance (p>0.05)<br><br>**<u>Psychological factors</u>**<br>NA | **<u>Physical factors</u>**<br>NA<br><br>**<u>Psychological factors</u>**<br>NA | **<u>Physical factors</u>**<br>NA<br><br>**<u>Psychological factors</u>**<br>NA |
| **Kuwahara et al. 2019** | 29 | Patients with LSS with leg pain and/or NC<br><br>M: F ratio= 17: 12<br><br>*Group1* (n=8): leg pain and LBP changes<br>Age ± SD = 72 ± 7 yo<br><br>*Group2* (n=12): leg pain changes only<br>Age ± SD = 69 ± 8 yo | **<u>Physical factors</u>**<br><br>***Pain***<br><br>**<u>Psychological factors</u>**<br>NA | **<u>Physical factors</u>**<br><br>***Pain***<br>**Leg pain changes – peak trunk tilt pre-effort (r=-0.455; p=0.044)**<br>Leg pain changes – velocity, peak trunk tilt post effort, peak thoracic tilt, peak lumbar tilt and peak pelvis tilt (r= [-0.206 - 0.286] ; p>0.05).<br>**Low back pain changes – peak lumbar tilt pre-effort (r=-0.508; p=0.022)**<br>**Low back pain changes – peak lumbar tilt post-effort (r=-0.503; p=0.024)**<br>**Low back pain changes – pelvis variation (r=0.506; p=0.023)**<br>Low back pain change - velocity, peak trunk tilt, peak thoracic tilt, changes in lumbar spine and peak pelvic tilt pre- and post-effort (r= [-0.274 - 0.343] ; p>0.05)<br><br>**<u>Psychological factors</u>**<br>NA | **<u>Physical factors</u>**<br>NA<br><br>**<u>Psychological factors</u>**<br>NA |
| **Minetama et al. 2020** | 71 | | **<u>Physical factors</u>**<br><br>***Objective measures***<br>**Daily step count – walking distance (r=0.338 ; p<0.05)**<br><br>**<u>Psychological factors</u>**<br>NA | **<u>Physical factors</u>**<br>NA<br><br>**<u>Psychological factors</u>**<br>NA | **<u>Physical factors</u>**<br><br>***Pain***<br>NRS (LBP, leg pain, leg numbness) – daily step count (r = [-0.200 - -0.003] ; p>0.05)<br><br>***Disability***<br>ZCQ (symptom severity, physical function) – daily step count ( r=[-0.218 - -0.215] ; p>0.05)<br><br>**<u>Psychological factors</u>** |

| | | | | | |
|---|---|---|---|---|---|
| | | | | | **PASS-20 (total score, cognitive anxiety, escape/avoidance, fear) – daily step count (r = [ – 0.352 – 0.338 ; p<0.05)**<br>PASS-20 (physiological anxiety) – daily step count (r = -0.229 ; p=0.055)<br><br>**HADS depression score – daily step count (r = – 0.245 ; p<0.05)**<br>HADS anxiety – daily step count (r = – 0. ; p=0.499)<br><br>PCS (total score, rumination, magnification, helplessness) – daily step count (r= [-0.189 - -0.137]; p>0.05)<br>TSK-11 – daily step count (r= -0.229 ; p=0.055) |
| **Nagai et al. 2014** | 11 | Patients with LSS with NC<br><br>M: F ratio= 8: 3<br>Age ± SD = 72.8 ± 5.5 yo | **Physical factors**<br><br>***Other objective measures***<br>**RMS at the cervical sensor – walking distance (r=-0.64; p=0.03)**<br>RMS at the lumbar sensor – walking distance (r=-0.55 ; p>0.05)<br>Stride frequency – walking distance (r=0.43 ; p>0.05)<br><br>**Psychological factors**<br>NA | **Physical factors**<br>NA<br><br>**Psychological factors**<br>NA | **Physical factors**<br>NA<br><br>**Psychological factors**<br>NA |
| **Özdermir et al 2015** | 108 | *Group 1* (n=54):<br>Patients with diagnosis of LSS by clinical findings and MRI<br>M: F ratio = 9: 45<br>Age ± SD = 68.06 ± 9.55 yo | **Physical factors**<br><br>***QoL***<br>**Walking capacity – SF-36 total score (r= 0.458; p<0.05)**<br>**Walking capacity – SF-36 physical health (r= 0.476; p<0.001)**<br>**Walking distance – SF-36 mental health (r= 0.319; p<0.05).**<br><br>**Psychological factors**<br>NA | **Physical factors**<br>NA<br><br>**Psychological factors**<br>NA | **Physical factors**<br>NA<br><br>**Psychological factors**<br>NA |
| **Pryce et al. 2012** | 33 | Patients with LSS with NC<br><br>M: F ratio = NA<br>Age ± SD = 68.5 ± 8.4 yo | **Physical factors**<br>NA<br><br>**Psychological factors**<br>NA | **Physical factors**<br>NA<br><br>**Psychological factors**<br>NA | **Physical factors**<br><br>***Pain***<br>**PA volume – back pain (intensity and function) and leg pain (function) (r = [-0.44 – -0.42] ; p<0.05)**<br>**PA duration – back pain (intensity and function) and leg pain (function) (r=[-0.42 – -0.38] ; p<0.05)** |

| | | | | | |
|---|---|---|---|---|---|
| | | | | | PA intensity – back and leg pain (intensity and function) (r=[-0.25 – -0.18] ; p>0.05)<br><br><u>Ambulatory behavior meaningful intensity:</u><br>**Bout length – back pain (r=-0.36 ; p<0.05)**<br>Bout length – back pain (function) and leg pain (intensity and function) (r=[-0.31 – -0.14] ; p>0.05)<br>**Maximum bout length – back pain (intensity and function) and leg pain (function) (r= [-0.43 – -0.29] ; p<0.05)**<br>Maximum bout length – leg pain intensity (r=-0.21 ; p>0.05)<br><br><u>Ambulatory behavior moderate intensity:</u><br>**Bout length – leg pain intensity (r=-0.35 ; p<0.05)**<br>Bout length – back pain (intensity and function) and leg pain (function) (r=[-0.30 – -0.23] ; p>0.05)<br>**Maximum bout length – leg pain intensity (r=-0.39 ; p<0.05)**<br>Maximum bout length – back pain (intensity and function) and leg pain (function) (r=[-0.34 – -0.30] ; p>0.05)<br><br><u>Sedentary behavior:</u><br>Duration, bout length and maximum bout length – back and leg pain (intensity and function) (r=[-0.24 – 0.26] ; p>0.05)<br><br>***Disability***<br>**PA (volume, intensity, duration) – ODI (r=[-0.52 – -0.35] ; p<0.05)**<br>**PA (volume, intensity, duration) – RMDQ (r=[-0.58 – -0.42] ; p<0.05)**<br>**PA (volume, intensity, duration) – DASH (r=[-0.57 – -0.49] ; p<0.01)**<br><br><u>Ambulatory behavior meaningful intensity:</u><br>Bout length – ODI (r=-0.30 ; p>0.05)<br>**Maximum bout length – ODI (r=-0.46 ; p<0.01)**<br>**Bout length and maximum bout length – RMDQ (r=[-0.50 – -0.38] ; p<0.05)**<br>**Bout length and maximum bout length – DASH (r=[-0.51 – -0.46] ; p<0.01)**<br><br><u>Ambulatory behavior moderate intensity:</u><br>**Bout length – ODI (r=-0.49 ; p<0.01)**<br>**Maximum bout length – ODI (r=-0.55 ; p<0.01)** |

| | | | | | |
|---|---|---|---|---|---|
| | | | | | **Bout length and maximum bout length – RMDQ (r=[-0.50 – -0.38] ; p<0.05)**<br>Bout length – DASH (r=-0.26 ; p>0.05)<br>**Maximum bout length – DASH (r=-0.38 ; p<0.05)**<br><br><u>Sedentary behavior behavior:</u><br>Duration, bout length and maximum bout length – ODI (r=[-0.14 – 0.30] ; p>0.05)<br>Duration, bout length and maximum bout length – RMDQ (r=[-0.10 – 0.32] ; p>0.05)<br>Duration, bout length and maximum bout length – DASH (r=[0.12 – 0.22] ; p>0.05)<br><br>***QoL***<br>**PA (volume, duration) – SF-36 (total, PH, MH, PF, BP)(r=[0.35 – 0.64] ; p<0.05)**<br>**PA (intensity) – SF-36 (total, PH, MH, PF)(r=[0.36 – 0.55] ; p<0.05)**<br>PA (intensity) – SF-36 BP (r=0.21 ; p>0.05)<br><br><u>Ambulatory behavior meaningful intensity:</u><br>**Bout length and maximum bout length – SF-36 (total, PH, MH, PF, BP) (r=[0.34 – 0.73] ; p<0.05)**<br><br><u>Ambulatory behavior moderate intensity:</u><br>**Bout length – SF-36 (total, PH, PF, BP) (r[=0.35 – 0.59] ; p<0.05)**<br>Bout length – SF-36 MH (r=0.32 ; p>0.05)<br>**Maximum bout length – SF-36 (total, PH, MH, PF) (r=[0.39 – 0.61] ; p<0.05)**<br>Maximum bout length – SF-36 BP (r=0.33 ; p>0.05)<br><br><u>Sedentary behavior behavior:</u><br>Duration, bout length and maximum bout length – SF-36 (total, PF, MH, PF, BP) (r=[-0.24 – 0.01] ; p>0.05)<br>**Bout length – SF-36 PF (r= -0.41 ; p<0.05)**<br>Bouth length – SF-36 (total, PH, MH, BP) (r=[-0.32 – -0.20] ; p>0.05)<br><br>**<u>Psychological factors</u>**<br>NA |
| **Quack et al. 2019** | 67 | Patients with degenerative LSS | **<u>Physical factors</u>**<br>NA<br><br>**<u>Psychological factors</u>** | **<u>Physical factors</u>**<br>NA<br><br>**<u>Psychological factors</u>** | **<u>Physical factors</u>**<br>NA<br><br>**<u>Psychological factors</u>** |

| | | | | | |
|---|---|---|---|---|---|
| | | M: F ratio = 34: 33<br>Age ± SD = 62.5 ± 11.7 yo | NA | NA | NA<br><br>***Anxiety, fear avoidance and depression***<br>**AAPI participation scale – DESC (r= -0.57 ; p<0.01)**<br>**RehaCAT lower extremity – DESC (r=0.43 ; p<0.01)**<br>**RehaCAT activities of daily living – DESC (r=0.45 ; p <0.01)**<br><br>**AAPI participation – TSK SF (r=-0.39 ; p<0.01)**<br>AAPI participation – TSK AA (r=-0.25 ; p>0.05)<br>**RehaCAT lower extremity – TSK SF (r=0.52 ; p <0.01)**<br>**RehaCAT lower extremity – TSK AA (r=0.45 ; p <0.01)**<br>**RehaCAT activities of daily living – TSK SF (r=0.43 ; p <0.01)**<br>**RehaCAT activities of daily living – TSK AA (r=0.44 ; p <0.01)** |
| **Schmidt et al. 2017a** | 300 | Patients with LSS<br><br>*Group1* (n=54): Patients with symptomatic LSS<br>Age ± SD = 76.9 ± 6.6 yo<br><br>*Group2* (n=246): Patients with no symptomatic LSS<br>Age ± SD = 77.1 ± 7.2 yo | **Physical factors**<br>NA<br><br>**Psychological factors**<br>NA | **Physical factors**<br>NA<br><br>**Psychological factors**<br>NA | **Physical factors**<br><br>***Objective measures***<br>**Trunk extensor muscle endurance, leg strength asymmetry and leg speed – SPPB score ($r^2$= 0.24)**<br><br>**Trunk extensor muscle endurance, knee flexion ROM and knee extension asymmetry – HGS ($r^2$= 0.35)**<br><br>**Leg strength – Chair Stand test ($r^2$= 0.19)**<br><br>**Psychological factors**<br>NA |
| **Sigmundsson et al. 2011** | 109 | Patients with a central spinal stenosis planned for surgery<br><br>M:F ratio = 53: 56<br>Age ± SD = 71 ± 10 yo | **Physical factors**<br><br>***MRI findings***<br>Estimated walking distance – MRI findings (r = -0.01 ; p>0.05)<br>Estimated walking distance – Multilevel spinal stenosis (r = -0.02 ; p>0.05)<br><br>**Psychological factors**<br>NA | **Physical factors**<br>NA<br><br>**Psychological factors**<br>NA | **Physical factors**<br>NA<br><br>**Psychological factors**<br>NA |
| **Thornes et al. 2018a** | 103 | Patients with a symptomatic and radiological verified central spinal stenosis<br><br>M:F ratio = 48: 55<br>Age ± SD = 71.3 ±7.3 yo | **Physical factors**<br><br>***Disability***<br>**Tandem walk – SSSQ functional scale (r= -0.29; p= 0.01)**<br>Tandem walk – SSSQ symptoms scale (r=-0.20 ; p>0.05) | **Physical factors**<br>NA<br><br>**Psychological factors**<br>NA | **Physical factors**<br><br>***Disability***<br>**30-s SS – SSSQ (symptoms scale: r= -0.29; p= 0.01), function scale: r=-0.44 ; p<0.01)**<br>**Stair climb – SSSQ (symptoms scale : r= 0.40; p=0.01, function scale: (r= 0.58; p=0.01)** |

| | | | | | |
|---|---|---|---|---|---|
| | | | **Psychological factors**<br>NA | | **OLS – SSSQ (symptoms scale : r= -0.33; p=0.01, function scale: r= -0.28; p=0.01)**<br><br>**Psychological factors**<br>NA |
| **Thornes et al. 2018b** | 62 | Patients with MRI-verified LSS<br><br>M: F ratio = 33: 29<br>Age ± SD = 71.2 ± 7.1 yo | **Physical factors**<br>NA<br><br>**Psychological factors**<br>NA | **Physical factors**<br>NA<br><br>**Psychological factors**<br>NA | **Physical factors**<br><br>***Disability***<br>**Mini-BESTest – SSSQ function scale (b= -0.1; p= 0.042**)<br><br>**Stability in Gait – ODI (b= -5.3; p=0.001)**<br>Mini-BESTest – ODI (b = -0.9 ; p>0.05)<br><br>**Psychological factors**<br>NA |
| **Tomkins-Lane, 2013** | 49 | Patients with LSS with NC or walking limitations<br><br>M: F= 25: 24<br>Age ± SD = 65.8 ±10.0 yo | **Physical factors**<br><br>***Pain***<br>**Walking capacity – years of back pain (r=0.29; p<0.05)**<br>**Walking capacity – years of leg pain (r=0.42; p<0.01)**<br>**Walking capacity – pre-walk leg VAS (r=-0.32; p<0.05)**<br>**Walking capacity – HUI Q8 (r=-0.35; p<0.05)**<br>**Walking capacity – HUI Q15 (r=-0.33; p<0.05)**<br><br>***Disability***<br>**Walking capacity – SSSQ symptom scale (r=-0.28; p<0.05)**<br>**Walking capacity – ODI (r=0.52; p<0.01)**<br><br>***QoL***<br>**Walking capacity – HUI score (r=0.38; p<0.05)**<br><br>***Other objective measures***<br>**Walking capacity – balance problems (r=-0.40; p<0.01)**<br><br>**Psychological factors**<br>NA | **Physical factors**<br>NA<br><br>**Psychological factors**<br>NA | **Physical factors**<br>NA<br><br>**Psychological factors**<br>NA |
| **Tong et al. 2007** | 48 | *Group 1* (n=24): Patients with lumbar spinal stenosis<br><br>M: F 14: 10<br>Age ± SD = 68.6 ± 6.8 yo | **Physical factors**<br><br>***Pain***<br>15-min walk test – PDI (β= -0.12 ; p>0.05)<br>7-day walking distance – PDI (β=0.23 ; p>0.05) | **Physical factors**<br>NA<br><br>**Psychological factors**<br>NA | **Physical factors**<br>NA<br><br>**Psychological factors**<br>NA |

| | | | | | |
|---|---|---|---|---|---|
| | | | ***Disability***<br>15-min walk test – QBPDS (β=-0.17 ; p>0.05)<br>7-day walking distance – QBPDS (β=0.05 ; p>0.05)<br><br>**<u>Psychological factors</u>**<br>NA | | |
| **Zeifang et al. 2008** | 63 | Patients with symptomatic LSS (N=63)<br><br>M:F ratio = 26: 37<br>Age (median) = 68 yo (13 IR) | **<u>Physical factors</u>**<br><br>***MRI findings***<br>**Walking capacity – MRI findings (tau b=-0.118; p=0.032)**<br><br>***Other objective measures***<br>Walking capacity – BMI (tau b= -0.194; p=0.025)<br>Walking capacity – functional status (tau b=0.225; p=0.011)<br><br>**<u>Psychological factors</u>**<br><br>***Anxiety and depression***<br>Walking capacity – CES-D (tau b = -0.053 ; p>0.05) | **<u>Physical factors</u>**<br>NA<br><br>**<u>Psychological factors</u>**<br>NA | **<u>Physical factors</u>**<br>NA<br><br>**<u>Psychological factors</u>**<br>NA |

M= male, F= female, SD = standard deviation, BMI = Body mass index, LSS= Lumbar Spinal Stenosis, NC= Neurogenic Claudication, ODI= Oswestry Disability Index, NRS= Numeric Rating Scale, SF-12= Medical Outcomes Short-Form 12, SPWT= Self-Paced Walking Test, QBPDS= Quebec Back Pain Disability Scale, SSSQ= Swiss Spinal Stenosis Questionnaire, SF-36= 36-item Short Form Health Survey, PH = Physical Health, MH = Mental Health, PF = Physical Function, BP = Bodily Pain, JOABPEQ= Japanese Orthopedic Association Back Pain Evaluation Questionnaire, ZCQ= Zurich Claudication Questionnaire, PASS-20 = Pain Anxiety Symptoms Scale, PCS = Pain Catastrophizing Scale, TUGT= Timed Up-and-Go Test, GDI= Gait Deviation Index, VAS= Visual Analog Scale, MRI= Magnetic Resonance Imaging, BDI= Beck Depression Inventory, EMG= Electromyography, RMS= Root Mean Square, HADS= Hospital Anxiety and Depression Scale, RMDQ = Roland-Morris Disability Questionnaire, DASH= Disability of the Arm, Shoulder and Hand questionnaire, ROM= Range of Motion, HGS= Habitual Gait Speed, DESC= Rasch-based Depression Screener, TSK= Tampa Scale of Kinesiophobia, AA= Activity Avoidance, SF= Somatic Focus, ADL=Activity of Daily Living, RehaCAT = Rasch-based RehaCAT-system, PI-G= Pain Interference Scale-German, SPPB= Short Physical Performance Battery, OLS= One Leg Stance test, 30-s SS= 30 seconds Sit to Stand, HSCL25= Hopkins symptom check list, HUI= Health Utilities Index Mark 3, PDI= Pain Disability Index, CES-D= Center of Epidemiological Studies Depression Scale, OR = Odds Ratio
